# High-Resolution Patterned Cellular Constructs by Droplet-Based 3D Printing

**DOI:** 10.1038/s41598-017-06358-x

**Published:** 2017-08-01

**Authors:** Alexander D. Graham, Sam N. Olof, Madeline J. Burke, James P. K. Armstrong, Ellina A. Mikhailova, James G. Nicholson, Stuart J. Box, Francis G. Szele, Adam W. Perriman, Hagan Bayley

**Affiliations:** 1 Department of Chemistry, University of Oxford, Oxford, OX1 3TA UK; 2 School of Cellular and Molecular Medicine, University of Bristol, Bristol, BS8 1TD UK; 3 Bristol Centre for Functional Nanomaterials, University of Bristol, Bristol, BS8 1TL UK; 4 Centre for Organized Matter Chemistry and Centre for Protolife Research, School of Chemistry, University of Bristol, Bristol, BS8 1TS UK; 5 Department of Physiology, Anatomy and Genetics, University of Oxford, Oxford, OX1 3QX UK

## Abstract

Bioprinting is an emerging technique for the fabrication of living tissues that allows cells to be arranged in predetermined three-dimensional (3D) architectures. However, to date, there are limited examples of bioprinted constructs containing multiple cell types patterned at high-resolution. Here we present a low-cost process that employs 3D printing of aqueous droplets containing mammalian cells to produce robust, patterned constructs in oil, which were reproducibly transferred to culture medium. Human embryonic kidney (HEK) cells and ovine mesenchymal stem cells (oMSCs) were printed at tissue-relevant densities (10^7^ cells mL^−1^) and a high droplet resolution of 1 nL. High-resolution 3D geometries were printed with features of ≤200 μm; these included an arborised cell junction, a diagonal-plane junction and an osteochondral interface. The printed cells showed high viability (90% on average) and HEK cells within the printed structures were shown to proliferate under culture conditions. Significantly, a five-week tissue engineering study demonstrated that printed oMSCs could be differentiated down the chondrogenic lineage to generate cartilage-like structures containing type II collagen.

## Introduction

The majority of tissues are composed of multiple cell types organized in the three-dimensional (3D) arrangements necessary for cell-cell communication and function. The fabrication of living tissues *in vitro* involves recapitulating this complex cytoarchitecture, which is difficult to do in a controlled fashion. However, the recent development of cell-printing technologies and 3D cell culture techniques have enabled the maturation of simple tissues from printed cellular constructs^1–4^, which are 3D organisations of cells of one or more type. Fabricated tissues with physiological form and complexity have been used in surgical implantation^5^, in toxicology^6^ and as tumour models^7^. Here we extend an approach previously used to 3D print aqueous droplet networks with tissue-like functionalities^8^ to the high-resolution patterning of living cells. Moreover, the low-cost method enables reproducible printing of 3D constructs with high cell viability at tissue relevant cell densities, with a low droplet dispensing volume of 1 nL, i.e. a droplet resolution that resides between those of traditional inkjet and valve-based bioprinters.

While bioprinting has advanced significantly over the last 15 years, the pursuit of morphological complexity and biological functionality in fabricated cellular constructs remains challenging^9^. Criteria relating to the printing process, including cytocompatibility, the resolution of cell placement and structural complexity, and the maturation of biologically active tissues, must all be addressed if printed tissues are to play a major role in regenerative medicine^2, 10^. To date, no single fabrication approach has addressed the gamut of design challenge for synthetic cellularised structures, however progress has been made by appropriating a range of 3D printing methodologies, including extrusion^4, 11–15^, laser-induced forward transfer^16^, and droplet-based ejection^17, 18^.

Extrusion-based bioprinters deposit a continuous filament of cell-laden hydrogels or cell spheroids^19^ onto a substrate in a layer-by-layer fashion^2^. Typically, the cellularised bioinks are composed of cells suspended in a biocompatible scaffold such as decellularised extracellular matrix^20^ or biopolymers^6, 21, 22^, for instance gelatin derivatives^12, 21, 22^ and alginate^21, 23^. By contrast, cell spheroids are typically deposited without a scaffold and can fuse together, reorganising into a single tissue during maturation^13, 24^. Advantageously, the lack of scaffold negates issues relating to scaffold biocompatibility and degradation^19^. A range of simple tissues or cellularised structures have been produced by extrusion-based bioprinting, including cartilage^20, 25^, bone^25^, muscle^25^ and adipose tissues^20^, 3D vasculature^12, 13^, aortic valves^21^ and beating cardiac cell assemblies^26^. Extrusion-based printers are ideally suited to the rapid manufacture of large structures (>1 cm^3^), and also have been employed to fabricate complex cell-free structures such as branched tubular networks in granular gel^27^. However, they can be deficient when applied to the high-resolution patterning of multiple cell types. High-resolution cell features require a small diameter nozzle, which greatly increases shear stress resulting in decreased cell viabilty^28^. Consequently, only in a few notable examples have viable cells been successfully extruded through nozzles of 200 μm diameter^12, 29, 30^ or narrower^22^. Laser-assisted bioprinting (LAB) is a nozzle-free system that avoids extrusion. In LAB, cell-containing microdroplets are ejected from the surface of a ribbon by pulsed laser irradiation of an underlying light-absorbing layer, and assemble on a collector substrate^31^. Although LAB initially focused on the 2D patterning of cells^31–33^, recent examples have established 3D architectures in the form of simple bilayers containing fibroblasts and keratinocytes as skin analogues^34^. However, the high-cost of laser-based systems and difficulties in constructing well-defined 3D architectures^2^ has prevented widespread uptake. Droplet-based bioprinters, such as inkjet^35, 36^ and valve-based technologies^37–39^, dispense cell-laden droplets from a nozzle by using thermal, pneumatic or sonic actuation, and were the first platforms used to pattern cells^36, 40^. Tissue fabrication by droplet methods has been limited to simple bone tissues^5^, fibro-cartilage interfaces^41^ and cartilage constructs^42^. Despite advantageous properties of inkjet bioprinters such as high cell viability^35, 36, 39^ and the sub-100 μm diameter of the dispensed droplets^40, 43^, there remain issues concerning clogging^35^, limited biopolymer compatibility^18, 44^ and sub-optimal cell density^35, 36^. While valve-based bioprinters have resolved clogging issues and have the advantage of being compatible with a range of viscous materials such as gelatin^41^, they have reduced droplet resolution compared to inkjet techniques^38, 39^, and the deposited droplets wet and spread on the printer substrate^39, 41^. Hybrid cell-printing techniques have emerged to overcome certain limitations of existing technologies. For example, extrusion can be supplemented with an electric field to reduce the damage to deposited cells caused by shear stresses within the dispensing nozzle, particularly at high pneumatic pressures. This has enabled fabrication at high-volume flow rates without compromising cell viability^23^. Furthermore, techniques employing two dispensing methods in tandem have afforded cellularised structures with non-cellular elements such as structural frameworks^20, 25^ or perfusable microchannels^45^. Even with these advances in bioprinting technologies, there are still only limited examples of 3D printed constructs that contain two or more cell types^12, 13, 21, 25^ and of these, only a few cases where the patterned cell features were less than 250 μm wide^34, 46^.

Here we present a bioprinting approach that complements existing methodologies by combining advantages of various fabrication routes into a single methodology to produce millimetre-scale constructs with defined cellular patterns at tissue-like densities. By broadening our existing approach for aqueous droplet printing in oil^8^, we demonstrate a high precision cell printing approach with a droplet resolution of 1 nL. The printed cellular constructs submerged in oil were subsequently encapsulated in a thin layer of gel for transfer to aqueous medium. By this means, we have generated patterned constructs from two populations of cells, with high-resolution 3D features including layers and channels under 200 μm in width, within robust cubic-millimetre-scale structures. The incorporated cells initially displayed high viability (90% average) and were present at tissue-like densities of 10^7^ cells mL^−1^, i.e., the same magnitude as the highest reported densities for a droplet-based bioprinting processes^43, 47^. Cell proliferation occurred over several days along with an overall increase in cell viability (>95% average). Significantly, ovine mesenchymal stem cells (oMSCs) in printed constructs responded to transforming growth factor-β3 (TGF-β3) and underwent differentiation to form cartilage-like structures. These data demonstrate that fundamental biological processes can remain intact after printing, which suggests that the approach presented here will be useful for complex tissue fabrication.

## Results

### 3D printing of cellular constructs

**Figure 1:**
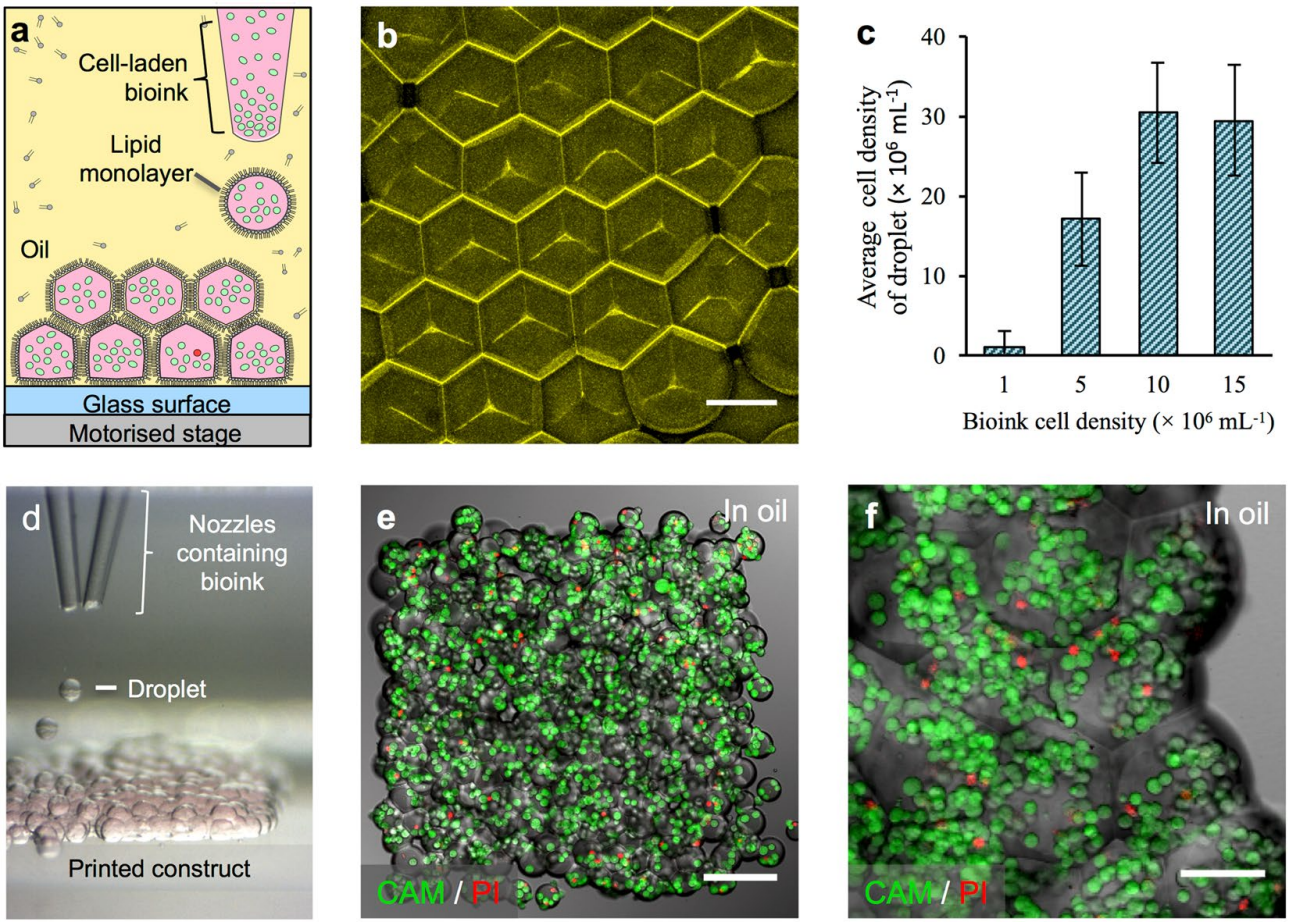
3D printing of cellular constructs. (**a**) Schematic of cell printing. The dispensing nozzle ejects cell-containing bioink droplets into a lipid-containing oil. The droplets are positioned by the programmed movement of the oil container. The droplets cohere through the formation of droplet interface lipid bilayers. (**b**) A confocal fluorescence micrograph showing droplet interface bilayers (stained yellow) within a cell-free printed construct (11 × 14 × 7 droplets). The bilayers were visualised by adding sulforhodamine-101 (~10 μM) to the print solution. (Scale bar = 100 μm). (**c**) Histogram showing the mean HEK-293T cell density in printed droplets under oil as a function of the cell density in the bioink. The cell density was calculated as the mean number of cells per droplet (*n* = 25) divided by the mean droplet volume. Error bars represent the compound error of droplet size variance and cell per droplet variance. (**d**) A bright-field micrograph of a patterned cell junction, containing two cell types, printed as successive layers of 1 nL droplets (*d* = 130 μm) ejected from two glass nozzles (*d* = ~150 μm). (**e**) A confocal fluorescence micrograph of a printed HEK-293T cellular construct (11 × 14 × 2 droplets) under oil. Live/dead cell staining was performed with calcein-AM (CAM, green) and propidium iodide (PI, red), respectively. Visible are approximately 700 cells at 4 × 10^7^ cells mL^−1^ with a viability of 85% (determined by manual cell counting). (Scale bar = 150 μm). (**f**) A high magnification, confocal fluorescence micrograph of a live/dead assay performed on an HEK-293T cellular construct (7 × 8 × 4 droplets) printed at a starting concentration of 1.5 × 10^7^ cells mL^−1^, with a mean occupancy of 38 cells per droplet equivalent to 3 × 10^7^ cells mL^−1^. Visible are some of the droplet boundaries. (Scale bar = 75 μm).

**Figure 2:**
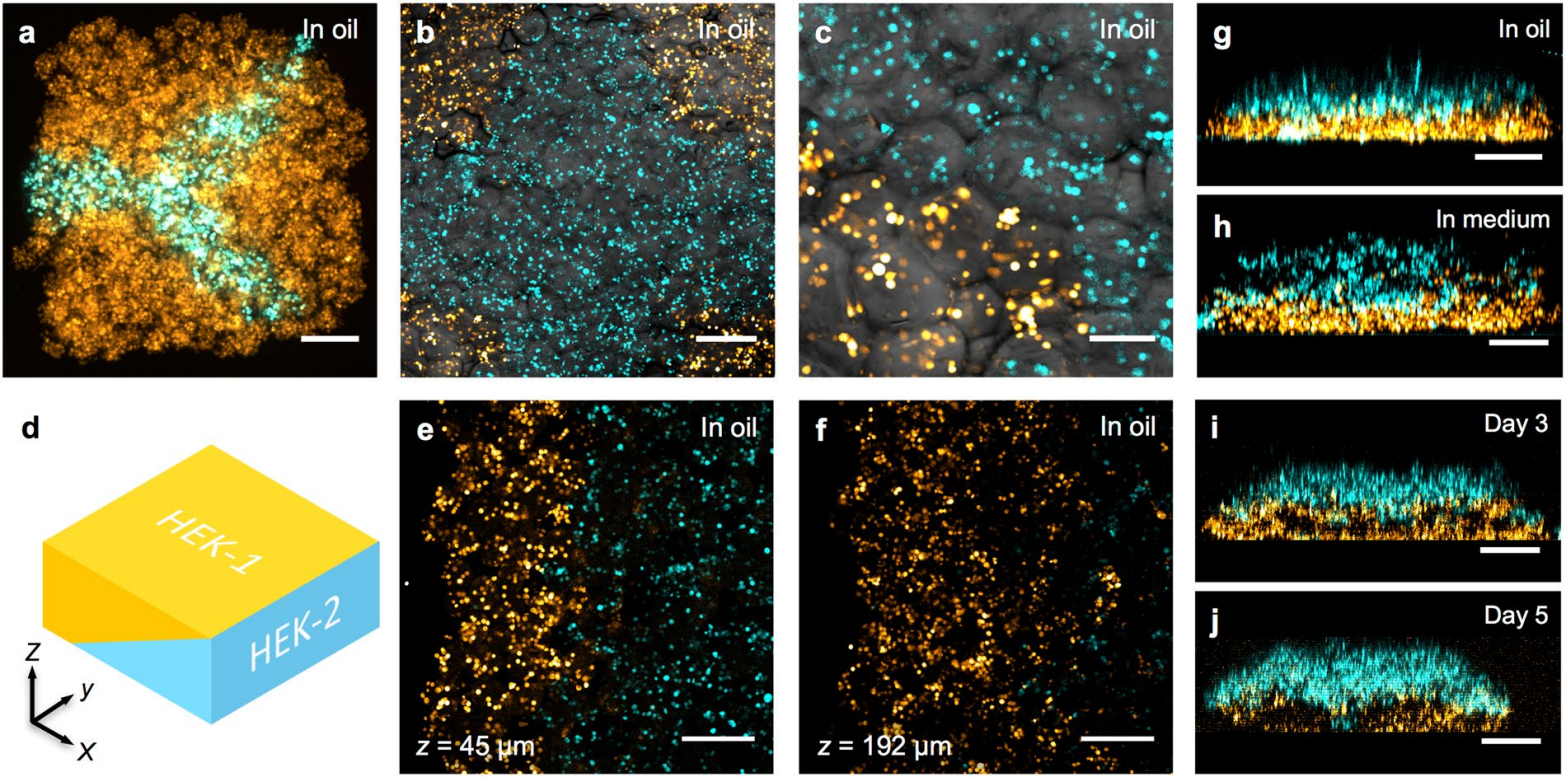
High-resolution patterning of two cell types. (**a**–**c, e,f**) Confocal fluorescence micrographs of printed cellular constructs in oil, immediately after printing. HEK-293 cells stained with Deep Red (DR) or Red CMPTX (RC) CellTracker™ dyes were false-coloured blue and yellow, respectively. (**a**) A Y-shaped structure within a square construct (8 × 9 × 4 droplets), with a mean feature width of 180 μm. (Scale bar = 200 μm). (**b**) A cruciform pattern of HEK-293 cells within a square construct (10 × 12 × 5 droplets). (Scale bar = 250 μm). (**c**) A high magnification image of the patterned HEK-293 cells in (**b**). (Scale bar = 100 μm). (**d**) A 3D model of a cuboidal cellular construct with an interface between two HEK populations (HEK 1, yellow; and HEK 2, cyan) at a diagonal in the *x*-*z* plane. (**e,f**) Partial cross-sections at fixed vertical positions (45 and 192 µm respectively) of a cellular construct (21 × 24 × 7 droplets) printed based according to the model in (**d**), showing both HEK populations. (Scale bars = 250 μm). (**g**–**j**) Side-on images of lamellar constructs, comprising CellTracker™ stained HEK-293 cells before and after phase transfer. The lower, DR-stained HEK-293 cell layers (yellow) were 3 droplets thick, while, the upper, RC-stained HEK-293 cell layers (blue) were 4 droplets thick (**g**,**h**) or 3 droplets thick (**i**,**j**). Images were recorded: (**g**) at day 0, in oil, immediately after printing; (**h**) immediately after transfer to culture medium; (**i**) on day 3 of culture, in medium and; (**j**) on day 5 of culture, in medium. (Scale bar = 250 μm).

**Figure 3:**
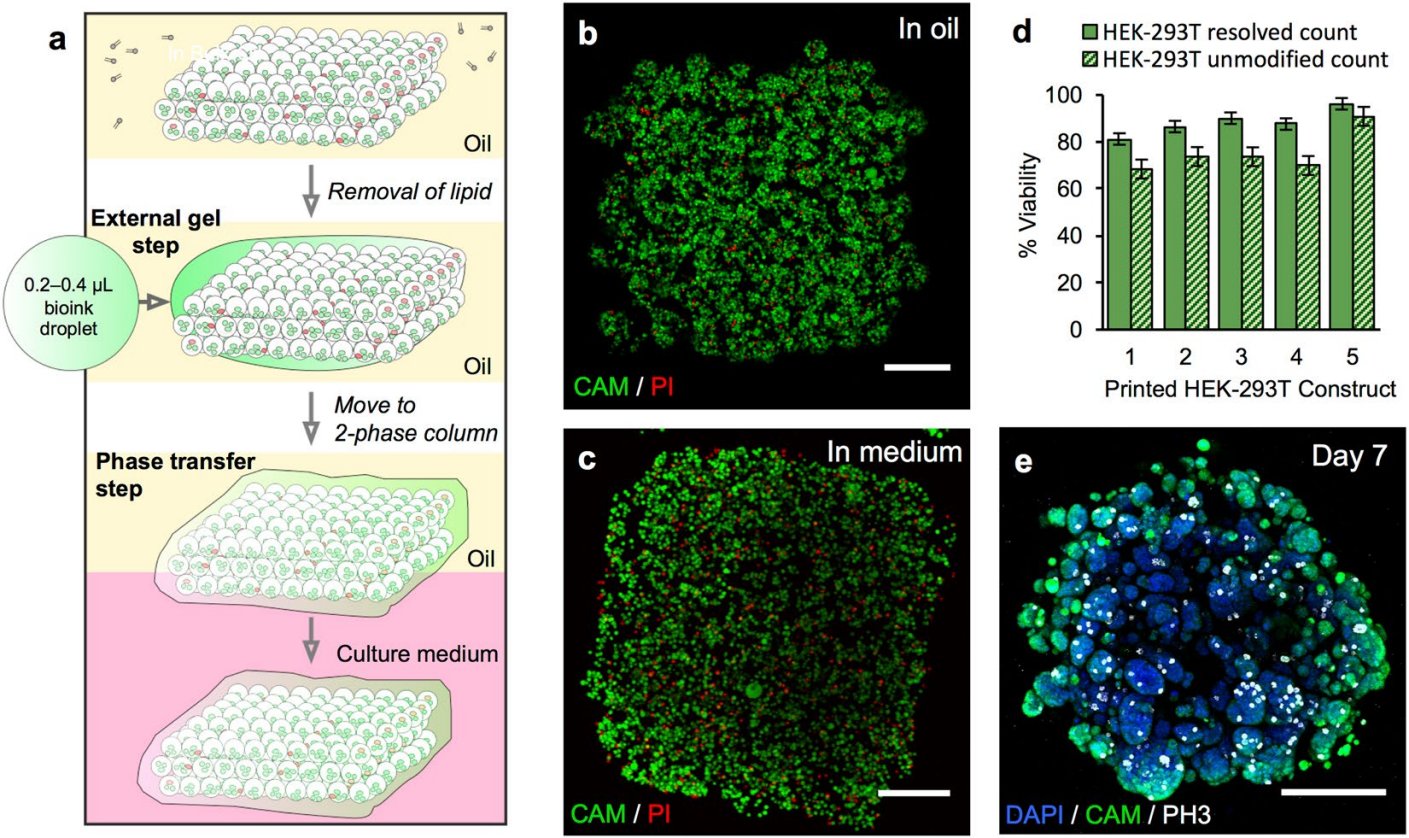
Phase transfer and culture of printed constructs containing HEK-293T cells. (**a**) Gel encapsulation of a printed construct and phase transfer. The printed cellular construct was gelled by standing at 4 °C for 20 to 25 min and the lipid in the oil was removed by washes with silicone oil AR20 at room temperature. The construct was then coated with a thin layer of cell-free bioink, which was gelled by standing at 4 °C for 20 to 25 min. The gelled construct was then transferred into the upper phase of an oil-culture medium two-phase system. The construct fell through the oil into the culture medium. (**b**) Image of a z-stack 3D reconstruction of live/dead-stained HEK-293T cells printed as a cuboid construct (7 × 8 × 4 droplets) immediately after printing under oil. The printed droplets had a mean density of 2.9 × 10^7^ cells mL^−1^ with a viability of 96%. (Scale bar = 200 μm). (**c**) Image of a z-stack 3D reconstruction of live/dead-stained printed HEK-293T cells after gel encapsulation and transfer to culture medium. (Scale bar = 200 μm). (**d**) Graph showing HEK-293T cell viability (including standard error of the mean) of five printed constructs at day 0 after transfer to culture medium. Viabilities were determined by using automated object counting, values of which were used either unmodified or resolved with respect to mean cell size. (**e**) Image of a z-stack 3D reconstruction of immunocytochemistry performed on a construct in culture medium at day 7: cell nuclei (DAPI, blue); cytoplasm of live cells (CAM, green) and; mitotic marker (phospho-histone H3 ICC, PH3, white). (Scale bar = 200 μm).

The high-resolution fabrication of cellular constructs was achieved by using a 3D printer previously employed to print aqueous droplets surrounded by lipid monolayers in oil^8^. Printer modifications allowed mammalian cells loaded inside the droplets to be patterned in a hydrogel-based bioink (Figs 1 and 2, Methods). The printed cellular constructs were then encapsulated in a thin layer of gel to allow transfer into aqueous medium (Fig. 3 and Supplementary Fig. 1). Human embryonic kidney (HEK-293T) cell derivatives (Supplementary Methods) were selected to evaluate the printing method, while oMSCs were used to investigate the differentiation capacity of cells in printed constructs.

HEK-293T cells or oMSCs were harvested and dispersed in a sterile bioink, which was kept at 37 °C prior to printing. The bioink contained serum-free defined cell culture medium, ultra-low-gelling-temperature (ULGT) agarose, fluorenylmethyloxycarbonyl (Fmoc) protected dipeptide gelators^48^ and routinely, type I collagen (Methods). The bioink was also compatible with other extracellular matrix proteins, and alternatively could be supplemented with fibronectin and laminin. In typical experiments, the cell-laden bioink (5 μL) was loaded into the glass bioprinter nozzle (Fig. 1a and d), which was subsequently immersed in a sterile blend (35:65 v:v) of undecane and silicone oil AR20 containing diphytanoyl phosphatidylcholine (DPhPC, Methods). The oil mixture had been optimized to allow aqueous droplets to descend by gravity within the oil phase, and to prevent freezing during the temperature-induced (4 °C) gelation performed in the gel encapsulation process (Supplementary Fig. 2). A programmed piezo-actuated impulse was used to eject cell-containing droplets (volume = 1 nL) from the nozzle into the oil (Fig. 1d). As the droplets sank (Fig. 1a), they acquired a DPhPC monolayer^8^, which allowed the subsequent formation of droplet-droplet interface bilayers (DIBs)^49^. Bilayer formation (Fig. 1b) conserved the print resolution by initially confining cells to limited volumes within the growing structure. Because the constructs were assembled in oil, the droplets packed in a hexagonal array. By contrast, when droplets are printed in air they flatten and the print resolution is reduced in the horizontal plane^39^. The constructs could be printed with high concentrations of cells without significant coalescence or loss of structural fidelity, which was attributed to the incorporation of Fmoc-dipeptides and the omission of fetal bovine serum (FBS) from the bioink (Supplementary Figs 3–5). To stabilise the constructs during gel encapsulation and phase transfer, the bioink was supplemented with agarose (1.0 to 1.2 w/v %), allowing the printed pattern to be preserved within solidified hydrogel (Supplementary Fig. 6). The presence of Fmoc-dipeptides also aided pattern retention by increasing the interfacial adhesion between printed droplets, and thereby producing a more cohesive structure (Supplementary Fig. 7).

Hundreds of nanolitre droplets were printed to give millimetre-scale constructs (Fig. 1e). Over the course of five minutes, structures with approximate dimensions of 1 × 1 × 0.5 mm could be printed. This was the typical size of printed constructs, but larger structures, up to nine times this volume were also fabricated (Supplementary Fig. 8). The printed droplets, had an average occupancy of between 1 and 38 cells depending on the bioink cell density (Supplementary Fig. 9). This resulted in a printed cell density in the range of 0.1 × 10^7^ to 3 × 10^7^ cells mL^−1^ (Fig. 1c), similar to the cell densities found in physiological tissues^50–52^. Typically, bioinks with starting concentrations of 1.0 × 10^7^ to 1.5 × 10^7^ cells mL^−1^ were employed as they achieved the highest printed cell densities (3 × 10^7^ cells mL^−1^), which arose due to sedimentation of the cells within the printer nozzle (Fig. 1f and Supplementary Fig. 9). This sedimentation enabled the printed cell density to be systematically varied by changing the initial cell density of the bioink (Supplementary Fig. 9). The small volume of bioink required for printing and the concentration of the cells by sedimentation makes our approach particularly appealing for the fabrication of tissues with scarce or high-value cells.

Live/dead cell staining performed on the day of fabrication (Fig. 1e and f) showed that the HEK-293T cell viability was 88 ± 5% ($\bar{x}$ ± s.d., *n* = 5) after printing. Similar viabilities were observed for non-printed HEK-293T cells cultured in blocks of gelled bioink and HEK-293T cells ejected as droplets in air directly into culture medium (Supplementary Fig. 10). Therefore, neither the bioink components nor piezo-induced stress was a primary cause of cell death. Indeed, the small fraction of dead cells that was observed was attributed to the periods spent away from ideal culture conditions (2 to 4 h total, during printing and processing) and to extended illumination during imaging by microscopy^53^ (Supplementary Fig. 11). Viability was increased to 90% or greater by returning printed constructs to the incubator between processing steps, minimising pH fluctuations during imaging by incorporating 20 mM HEPES buffer and imaging cellular constructs only once (Supplementary Methods).

### Fabrication of patterned cellular constructs

In addition to constructs printed from one type of cell, constructs containing two types of cell were printed in various high-resolution patterns (Fig. 2). These materials contained either separately labelled populations of the same cell line (*e*.*g*. HEK-293T stained with either Deep Red or Red CMPTX CellTracker™ dyes) or different cell types (osteoblasts and chondrocytes, see below). For example, HEK-293T cells were patterned as a Y-shaped junction within a cuboid (Fig. 2a), demonstrating the ability to fabricate features at a resolution of 1 to 2 droplet diameters (feature width 180 ± 60 μm, $\bar{w}$ ± s.d., 14 measurements). HEK-293/YFP and HEK-293/CFP cells were patterned into structures (Fig. 2b and c), which incorporated a cross motif (feature width 425 ± 73 μm, $\bar{w}$ ± s.d., 12 measurements) within a cuboid, with discrete cell types in the cruciform and corner segments. Constructs containing more complex 3D patterns of cells were also fabricated. For example, a 3D junction between HEK-293T and HEK-293/CFP cells was printed within a cuboidal structure (Fig. 2d, e and f, and Supplementary Figs 12 and 13). Here, the interface between the two cell populations was at a diagonal in the vertical (*x*-*z*) plane (Supplementary Figures 12 and 13) with a measured angle of 21 ± 8° with respect to the print surface ($\bar{x}$ ± s.d., 12 measurements, Supplementary Methods). Additionally, a high-resolution interdigitated interface between the two cell populations was achieved by patterning cell-laden droplets in an interdigitated fashion in the *x*-*y* plane (Supplementary Figure 12).

Finally, lamellar structures (Fig. 2b and e–h) were fabricated from two types of stained HEK-293 cells. Each lamellar construct had a final thickness of ~400 μm and comprised two layers of cells, with each layer 3 to 4 droplets thick. In this instance, droplet printing allowed precise and repeatable fabrication of lamellar structures with well-defined external dimensions (1.1 × 1.1 × 0.4 mm) and internal layer thicknesses (lower layer: 160 ± 26 μm and upper layer: 200 ± 17 μm, $\bar{x}$ ± s.d., *n* = 3 constructs). The ability to print spatially organised structures with predefined lineages was also possible. oMSC-derived osteoblasts and primary chondrocytes were suspended in separate bioinks and printed in a layered, 3D geometry as a model of a 3D osteochondral interface (Supplementary Fig. 14).

### Transfer of cellular constructs to medium for culture

To transfer the 3D printed cellular constructs from oil to culture medium without loss of pattern fidelity, a stabilization process was developed (Fig. 3). The constructs were cooled from ambient temperature and kept at 4 °C for 20 to 25 min to trigger gelation of the agarose in the bioink (Supplementary Fig. 1). After cooling, the constructs were encapsulated in additional ULGT-agarose to confer further stability during the phase transfer procedure (Methods). To mediate gel coating, the lipid of the print oil was first diluted to ~15 μM, *i*.*e*. ~1% of its original concentration, by repeated silicone oil (AR20) washes at room temperature (Methods). Afterwards, a droplet (0.2 to 0.4 µL) of the bioink (containing agarose, but without cells) was pipetted onto the external surface of the construct and solidified by standing at 4 °C for 20 to 25 min to produce a robust veneer (Fig. 3a and Supplementary Fig. 1). The calculated thickness of a veneer formed from the added bioink was 45 to 81 μm, assuming that it spreads evenly over the printed construct (Supplementary Methods). Confocal micrographs showed that the average veneer thickness at the vertical surface of the construct was 32 ± 9 μm (*n* = 3 constructs, Supplementary Methods). For phase transfer^54^, a coated cellular construct was loaded into a truncated pipette tip (20 μL) and transferred into the upper phase of an oil-above-culture-medium two-phase system. The cell construct fell into the aqueous phase by gravity sedimentation, shedding the bulk oil phase. The gelation process had a negligible impact on the organization of the printed structures as demonstrated for the printed lamellar constructs (Fig. 2e,f). The external dimensions were unchanged and the discrete cell layers were retained after phase transfer. The transfer process was performed successfully with more than 100 cuboidal cellular constructs (Supplementary Fig. 15).

The ability to deliver live/dead stain to cellular constructs in culture medium demonstrated the penetration of small dye molecules into the structures (Fig. 3c), which indicated that the lipid bilayers between adjacent droplets had been disrupted as a result of the gelation and oil washing steps. However, the structural role of the bilayers was no longer required at this point and their breakdown enabled the ingress of nutrients necessary for cell growth and the maintenance of physiological osmolarity.

After transfer to culture medium, the printed constructs were cultured and assayed for cell viability and proliferation over one week (Supplementary Fig. 16) with a change of culture medium every 2 to 3 d. Live/dead assays performed on the HEK-293T constructs revealed cell viabilities in excess of 80% immediately after phase transfer (Fig. 3d) and rising to >95% on days 3 and 7 (*n* = 4, Supplementary Table 4). DAPI nuclear staining showed that the cell population within the HEK-293T constructs exhibited cell division over the seven-day period. The average number of visible DAPI stained cells was observed to increase from 3,400 ± 1,600 cells ($\bar{x}$ ± s.d., *n* = 4) on day 3 to 11,600 ± 5,100 cells ($\bar{x}$ ± s.d., *n* = 4) on day 7 (Supplementary Fig. 17). Immunocytochemical (ICC) staining (Methods) showed dividing HEK-293T cells throughout the entire printed structures at day 3 and 7 of culture (Fig. 3e and Supplementary Figs 18 and 19), with 2 to 8% of the cells (*n* = 8) displaying phospho-histone H3 signals, characteristic of the G2/M transition (Supplementary Fig. 17). Furthermore, after 7 d, HEK-293T constructs had evolved from structures with individually distinguishable cells compartmentalised within printed droplets (Fig. 3b) into structures dense with multiple cell aggregates up to 140 μm in width (Fig. 3e), indicating that cells had proliferated and outgrown the confines of the printed droplets. Similar behaviour was also found in lamellar cellular constructs (Fig. 2e–h), which retained printed patterns of discrete cell layers after 5 d of growth.

### Development of printed stem cells

**Figure 4:**
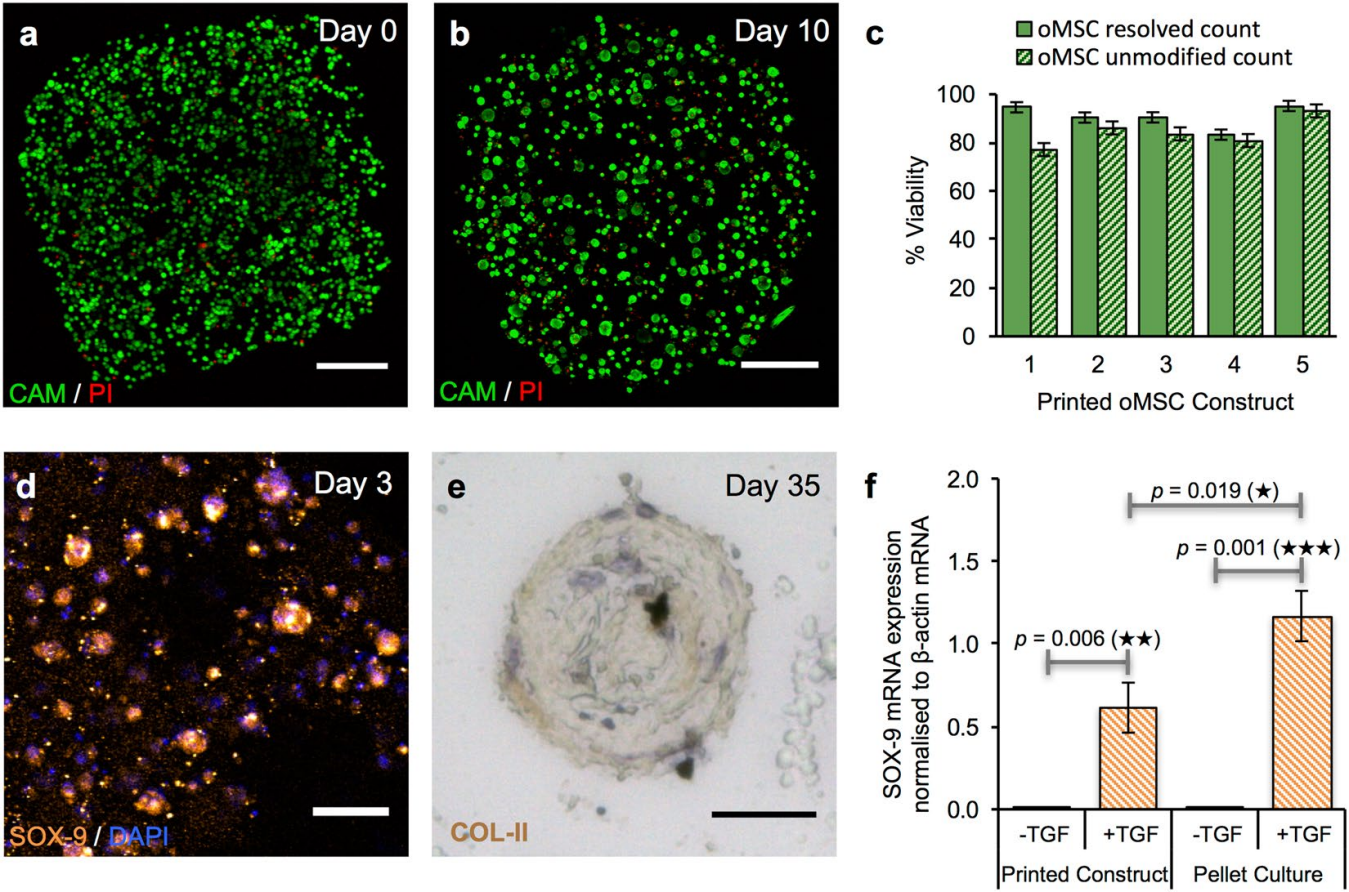
Growth and differentiation of printed oMSCs. (**a**,**b**) Image of a z-stack 3D reconstruction of live/dead-stained printed oMSCs: (**a**) immediately after printing and; (**b**) after 10 days in culture with the TGF-β3 supplement. (Scale bars = 250 μm). (**c**) Graph of oMSC viabilities (including standard error of the mean) for five printed constructs immediately after transfer to culture medium. Viabilities were determined by using automated object counting, values of which were used either unmodified or resolved with respect to mean cell size. (**d**) Confocal fluorescence micrograph of immunocytochemistry performed on a printed oMSC construct after 3 days of culture with a TGF-β3 supplement: SOX-9 (orange); nuclei (DAPI, blue); cytoplasm of live cells (CAM, calcein-AM, green). (Scale bar = 50 μm). (**e**) High-magnification micrograph of immunohistochemistry performed on a printed oMSC construct after 35 days of culture with TGF-β3 supplement; type II collagen (diaminobenzidine tetrahydrochloride (DAB), brown); nuclei (hematoxylin QS, blue). (Scale bar = 25 μm). (**f**) Digital PCR measurements of SOX-9 mRNA expression in printed oMSC constructs (*n* = 22) and oMSC pellet cultures (*n* = 24) after 7 days in chondrogenic medium with or without supplementation of TGF-β3. Each printed and pellet sample was replicated 4 to 6 times from four oMSCs sources, each extracted from a different sheep. SOX-9 expression was normalised to an endogenous β-actin control. Error bars represent standard deviations. Differences were tested by using a paired *t*-test, with two-tailed *p* values < 0.05 considered significant.

Having successfully printed and cultured constructs of HEK-293T cells, a robust mammalian cell line, the methodology was applied to mesenchymal stem cells (Fig. 4), which have therapeutic potential in regenerative medicine^17^. The viability of printed oMSCs immediately after printing (Fig. 4c) was 91 ± 4% ($\bar{x}$ ± s.d., *n* = 5), which was similar to the high post-print viabilities observed for HEK-293T cells (Fig. 3d). The ability of the printed oMSCs to differentiate was investigated by introducing TGF-*β*3, a chondrogenic growth factor, into the culture medium (Methods, Supplementary Fig. 20). ICC performed at days 3 and 7 revealed expression of the early chondrogenic transcription factor SOX-9 in oMSC constructs exposed to TGF-*β*3 (Fig. 4d,e and Supplementary Fig. 21), but not in untreated network controls (Supplementary Fig. 22). SOX-9 immunofluorescence was present in all cells in treated constructs. Tissue-wide expression of SOX-9 protein was also observed in a positive control of engineered cartilage, formed over 35 d using oMSCs seeded within a polyglycolic acid scaffold (Supplementary Fig. 22). Digital polymerase chain reaction (dPCR) analysis was used to quantify SOX-9 mRNA, normalised to the endogenous expression of *β*-actin mRNA, in printed constructs (*n* = 22) taken from four oMSC sources (Fig. 4f and Supplementary Figure 23). After 7 d, dPCR showed upregulation of SOX-9 mRNA for the treated printed constructs only. The upregulation of SOX-9 mRNA expression was lower for the treated printed constructs (0.61 ± 0.15, $\bar{x}$ ± s.d., *n* = 22) than for treated pellet cultures (1.17 ± 0.15, $\bar{x}$ ± s.d., *n* = 24), the gold standard for chondrogenesis^55^ (Fig. 4f). This was unsurprising given that the reduced intercellular space in cell pellets is commonly used to maximise the juxtacrine signalling (*e.g. via* N-cadherin, N-CAM) required for optimal chondrogenesis. Significantly, within 3 d, the oMSCs in the printed constructs treated with chondrogenic factors underwent spontaneous re-organisation to form spherical aggregates, 93% of which were between 20 and 60 μm in diameter (Fig. 4b, Supplementary Figs 20 and 24). This process resembled cellular condensation, a critical stage preceding chondrogenesis^56^ and was also observed in the untreated cellular constructs, however, the distribution of aggregate sizes was different (Supplementary Fig. 24). Cell aggregates within treated constructs remained a similar size over 10 d during early stage chondrogenesis, whereas untreated constructs contained fewer aggregates, with the majority of the cells present within larger aggregates that were >100 μm wide (Supplementary Figs 20 and 24). Advantageously, our approach distributes cells homogenously throughout the construct, which is in contrast to the inhomogeneous cell distributions often observed after seeding scaffolds for conventional tissue engineering^57^.

Following the preliminary chondrogenesis results, the ability to engineer tissues with properties of hyaline cartilage from printed oMSCs was investigated over a five-week period. Printed oMSC constructs (*n* = 13) were cultured for 35 days in a differentiation medium containing ascorbic acid, dexamethasone, TGF-*β*3 and insulin, a typical protocol for stem cell cartilage engineering^58^. During culture, the majority of printed oMSCs condensed into ~60-µm-wide aggregates, with each construct comprising 20–30 aggregates evenly distributed across the 1 × 1 × 0.5  mm printed structure (Supplementary Fig. 25). Immunoperoxidase staining of the printed constructs revealed the presence of secreted type II collagen, a key component of the extracellular matrix (ECM) of hyaline cartilage (Fig. 4e and Supplementary Fig. 25). Interestingly, the printed constructs exhibited negligible expression of type I collagen, a major component in fibrocartilage, but unwanted in engineered hyaline cartilage. These results demonstrate that printed cellular constructs can be maintained in long-term culture (35 days) without loss of structural fidelity and that the component cells can undergo differentiation and matrix secretion to produce tissue-like structures.

## Discussion

In this study, we have presented a method for the reproducible 3D printing of several types of mammalian cell with a high degree of spatial control. Our device can print droplets (1 nL/130 μm diameter) containing highly viable cells at a finer resolution of deposition than the majority of extrusion bioprinters (commonly ≥200 μm diameter nozzles^12, 13, 20, 21, 25, 30, 59^) and valve-based bioprinters (commonly ≥10 nL droplets^38, 39, 41, 60, 61^). The resolution approaches but does not exceed traditional inkjet printers (5 to 50 μm droplet diameter^40, 44^) but advantageously, our printed constructs contain cells at tissue relevant densities (3 × 10^7^ cells mL^−1^), which is unattainable by inkjet methods. The resolution of printing is enhanced because cells are maintained within the droplet during fabrication. Furthermore, the oil phase provides a structural support during printing that prevents the droplets from flattening, in contrast with droplet-based bioprinting performed in air which consequently has low *x*-*y* resolution^38, 39, 41^. The ability to repeatedly generate low volume droplets and precisely place them has enabled the high-resolution patterning of two cell populations. Cells were patterned as lamellae, diagonal plane interfaces and arborised junctions within millimetre-scale cuboidal constructs with feature resolutions of <200 μm. The patterning of two cell types at resolutions this fine has only been achieved previously by laser-assisted bioprinting^34, 46^. Specifically, skin-mimicking lamellar constructs comprising layers of keratinocytes and fibroblasts of <250 µm thicknesses have been generated. Other techniques, such as extrusion-based cell printing, have also patterned two cell types by depositing spheroids^13^ or cell-laden hydrogels^12^, but these approaches have only achieved features of ≥250 µm. Cellular constructs were reproducibly fabricated by using our high-resolution printer and transferred to culture medium without loss of structural fidelity. This high-resolution patterning method is therefore a useful tool for the fabrication of complex cellular constructs. By contrast, it is not possible to manufacture similar structures by either casting hydrogel-based solutions or cellular seeding of scaffold as this would produce structures with inhomogeneous cell distributions^57^. Currently, the constructs are limited to two cell types only, but in the future, the printer could be adapted to include a multi-nozzle dispenser increasing the number of patternable cell types.

The average viabilities of both HEK-293T cells and oMSCs was approximately 90% immediately after phase transfer. This high cell viability after printing is comparable to other state-of the art cell printing methods, such as extrusion printing of a gelatin composite hydrogel (>95% for 3T3 cells)^25^, droplet printing of a supramolecular polypeptide-DNA hydrogel (99% for At20 cells)^39^ and laser-based cell printing techniques (90 to 98%)^33^. The printed constructs were cultured for up to 35 days and the component cells remained viable and able to proliferate. Moreover, printed mesenchymal stem cells remained responsive to external cues and growth factors, as shown by a differentiation study in which TGF-*β*3 induced the upregulation of SOX-9, an early chondrogenic transcription factor, and the secretion of type II collagen, a key ECM component of hyaline cartilage. The retention of biological properties by the cells was attributed to the gentle printing method, the bioink formulation and the size of the constructs, which were thin enough not to be hindered by nutrient and oxygen diffusion limits. Thus, the printing approach produces millimetre-scale cubic constructs with patterned cells that are biologically active. Cellular constructs of this size are desirable for high-throughput screening assays as they size-compatible with 96-well plates. Furthermore, the production of tens or potentially hundreds of mm-scale constructs could be achieved during a single print run.

For the manufacture of cellular constructs for surgical implantation centimetre-scale structures^25^ will be required. In the present work, only microliter volumes of cells were printed which is advantageous for high-value cells, such as low-yield primary cell samples or gene-edited cell-lines, however, this print volume can be expanded to hundreds of microliters and potentially millilitre quantities, therefore, allowing printing on a centimetre-scale. However, the current encapsulation method, which is necessary for stabilising the structure during phase transfer, would likely need to be adapted to convey the required rigidity to larger constructs. In addition, the fabrication of centimetre-scale structures in a practicable manner would require the rate of printing to be significantly increased by generating droplets at higher frequencies (up to 20 Hz is feasible with the current hardware) and the simultaneous use of multiple droplet generators. Alternatively, larger structures could be attained by assembling mm-scale printed structures in a modular fashion and allowing the cells to mature into a single tissue over time. Regardless of the fabrication method, any constructs thicker than the oxygen diffusion limit (100 to 200 μm)^62^ must include microchannels or vasculature to sustain the tissue. Potentially, this might be achieved by incorporating artificial channels formed from sacrificial gel droplets. Similar approaches have been used to make extrusion-printed vascularized tissue^30^.

The versatility and robust nature of our approach provides a new set of tools for bottom-up tissue engineering at a low cost. Our droplet-based bioprinter is relatively inexpensive, costing approximately £7,500 to set up. The device is therefore at the low-cost end of commercial bioprinters, which are valued from ~£5,000 (*e*.*g*. Inkredible, CELLINK) to £160,000 (*e*.*g*. 3D Bioplotter, EnvisionTEC). Furthermore, the capability of the printer has allowed the fabrication of patterned cellular constructs with high-resolution features (<200 um) and with cells present at high viability (90% average) and tissue-like densities (10^7^ cells mL^−1^). Tissue-like structures can develop from these cellular constructs, as demonstrated by oMSCs that were differentiated after printing and then secreted type II collagen, a key ECM component of hyaline cartilage. We envisage that the high-resolution printing technique presented here will continue to be improved and may be used in a hybrid manner by combining with other technologies. For example, combining with an extrusion-based system may allow the rapid fabrication of clinical-scale tissues with complex 3D cellular features unattainable by extrusion-based or droplet printing alone. Alternatively, the printer may be used alone to print specialised cells, such as induced pluripotent stem cells^63^, into patterned cellular constructs which, after maturation, mimic, simplify or elaborate upon a range of natural tissues. In addition to their potential use in regenerative medicine, printed cellular constructs will impact near-future technologies in toxicology and disease modelling.

## Methods

### Bioink Preparation

Prior to cell addition, the bioink consisted of an 8:1 v:v mixture of ULGT-agarose solution to Fmoc-dipeptide solution, with or without collagen (Supplementary Methods). Typically, ULGT-agarose (13 to 15 mg mL^−1^, Sigma-Aldrich) dissolved in culture medium was heated to 65 °C. To this was added a 1:1 v:v mixture of 10 mM Fmoc-diphenylalanine (Fmoc-FF, Bachem) and 10 mM Fmoc-isoleucine-glycine (Fmoc-IG, Bachem) in Milli-Q water. The mixture was kept at ~50 °C until the addition of collagen or cells, before which it was cooled to 37 °C. When collagen was used, the bioink comprised a 9:1 v:v mixture of the agarose with Fmoc-dipeptide solution to type I collagen suspended in phosphate-bufferd saline (PBS). Bovine collagen (Life Technologies) was typically added to the bioink as a 3 mg mL^−1^ concentration solution producing a bioink with a final collagen concentration of 15 μg mL^−1^. The bioink was sonicated immediately prior to cell addition (5 min, 37 °C, 40 kHz) in a Branson 2800 ultrasonic bath. The solution was sterilized by irradiation with UV (15 min, 365 nm) prior to the addition of cells at 4.5 cm from an UV LED (Eclipse-M365L2-C5, Nikon) controlled by an LED driver (LEDD1B, Thorlabs) set to half power. The cells to be printed were harvested and then centrifuged (3 to 5 min, 300 to 500 × g). The pellet was resuspended in bioink (100 to 200 μL) at a density of 0.5 × 10^7^ to 1.5 × 10^7^ cells mL^−1^, most often 1.5 × 10^7^ cells mL^−1^. Bioink solutions were used on the day they were made.

### 3D Bioprinter

Cellular constructs were fabricated by using a droplet-based 3D printer developed in previous work^8^, with modifications of selected components (see Supplementary Methods). In brief, the printer consisted of a static piezoelectric actuated droplet generator, which ejected droplets from an oil-immersed nozzle into a lipid-in-oil bath. The bath rested on a motorised stage (PatchStar micromanipulator, Scientifica), which travelled along three orthogonal axes with a positional accuracy of 20 nm.

### 3D Printing

The printer components were first sterilised and the printing nozzles were then treated with oxygen plasma (Supplementary Methods). Bioink (~4 μL) was suction loaded into the printer nozzle beneath a hexadecane oil (Sigma-Aldrich) plug (1 to 2 μL). The nozzle was submerged in the oil chamber, which contained 200 to 250 μL of a 35:65 v:v mixture of undecane (Sigma-Aldrich) to silicone oil AR20 (Sigma-Aldrich) containing 1.2 to 1.5 mM 1,2-diphytanoyl-sn-glycero-3-phosphocholine (DPhPC, Avanti Polar Lipids). A voltage pulse, which reproducibly ejected single, monodisperse droplets, was determined and used for automated printing along with other print parameters (Supplementary Methods). Constructs were automatically printed by ejecting droplets between 120 and 160 μm diameter (typically 130 μm), according to 2D digital “maps” (with dimensions of *x* × *y* pixels) for a selected number of layers (*z*). Constructs of a single cell type were printed as layers of droplet sheets with a range of dimensions from 7 × 8 to 11 × 14 droplets. For phase transfer experiments, 4 layers of droplets was the usual thickness. For the lamellar, Y-shaped and cruciform patterned constructs, two cell-laden bioinks were printed successively with a single droplet generator, which was cleaned and reloaded after printing each cell type. Lamellar constructs comprising two discrete cell layers were printed as lower sheets (typically 7 × 9 droplets) for 3 layers followed by upper sheets (typically 6 × 7 droplets) for 3 to 4 layers. The Y-shaped and cruciform patterned constructs were printed with external dimensions of 10 × 12 and 8 × 9 droplets, respectively, for 2 to 6 layers. By contrast, the cell junction with a diagonal interface in the vertical (*x*-*z*) plane was printed as 7 layers of 21 × 24 droplets with two discrete bioinks by using two droplet generators in tandem.

### Gelation and Phase Transfer

The printed constructs were gelled after printing at ambient temperature by maintaining them at 4 °C, for 20 to 25 min. The lipid in the print oil was subsequently diluted to ~15 μM at ambient temperature, by removing half or more of the oil from the print container (leaving ~100 μL), adding silicone oil AR20 (200 μL), and then removing the oil mixture (200 μL). The silicone oil addition and mixed oil removal step was repeated three more times. The gelled construct under oil was coated with a droplet of ULGT-agarose solution (previously heated to 50 °C) ejected from a pipette (0.2 to 0.4 μL, 13 to 15 mg mL^−1^). The encapsulated printed constructs were then gelled again (4 °C, 20 to 25 min) and either phase transferred immediately or stored in a high humidity environment. Phase transfer was performed with oil-medium interfaces prepared in individual wells of a sterile 8-well microscope slide (II Chamber Slide™, Lab-Tek™). The oil was a 3:1 v:v mixture of hexadecane to mineral oil (M3516, Sigma-Aldrich, ~250 μL) above culture medium (~300 μL). The two-phase columns were incubated for ≥15 min in a cell incubator before use (37 °C, 5% CO_2_). Gelled coated cellular constructs were transferred into the upper oil phase and fell through the interface. After transfer, the oil phase was discarded and an additional 300 μL of culture medium was added.

### Culture of Printed Cells

The cellular constructs were grown after phase-transfer in microscope slides containing culture medium (~600 μL per well) over a period of up to ten days in a cell incubator (37 °C, 5% CO_2_). The culture medium was exchanged every 2 to 3 days as described in Supplementary Methods.

### Live/Dead Assay

Live/dead staining of cellular constructs was performed by using a calcein-AM (CAM) dye (Cambridge Biosciences Ltd) in conjunction with propidium iodide (PI, Sigma Aldrich). A dye solution of 0.05 mM CAM and 0.05 mM PI (Supplementary Methods) was added to the cell-laden bioink prior to printing or to the culture medium of printed constructs at a final concentration of ~5 μM for each component. Constructs were imaged by confocal fluorescence microscopy (Leica SP5, Supplementary Methods).

### Constructs Containing Two Cell Populations

CellTracker™ dyes (Life-Technologies), Red CMPTX (RC) and Deep Red (DR), were used to fluorescently stain cells. Prior to printing, the cells were suspended in serum-free culture medium containing either 5 μM RC or 1 μM DR (Supplementary Methods). The cells stained were: HEK-293T, CFP expressing HEK-293, primary chondrocytes and oMSC-derived osteoblasts. Printed constructs were imaged by fluorescence confocal microscopy (Leica SP5) and wide-field light microscopy (Leica DMI 8).

### Immunocytochemistry of Printed Constructs

Immunocytochemistry was performed on cellular constructs fixed in paraformaldehyde (Supplementary Methods). Primary antibodies were: 0.25% v/v rabbit anti-phospho-histone H3 (Merck Millipore: 06-570) or 0.67% v/v rabbit anti-SOX-9 (Merck Millipore: AB5535). Secondary donkey antibodies conjugated to Alexa Fluor 568 or 647 (Invitrogen: A10042 and A31573) were used with the SOX-9 and phospho-histone H3 antibodies, respectively. The immunostained constructs were imaged by confocal fluorescence microscopy (Zeiss LSM 710, Supplementary Methods).

### Immunohistochemistry of Printed oMSC Samples

Printed oMSC constructs and oMSC pellets were cultured in differentiation medium with TGF-β3 for 35 d. These samples were harvested and fixed in 4% v/v paraformaldehyde, embedded in paraffin and sectioned as 4 µm thick slices (Supplementary Methods). Samples were rehydrated by sequential immersions of 2 min duration in xylene, 100%, 90%, 80% and 70% (v/v) ethanol and deionized water. Immunohistochemistry was performed on sections with rabbit anti-type I collagen (Abcam: AB34710) and rabbit anti-type II collagen (Abcam: AB34712) primary antibodies, and polymeric HRP-conjugated anti-rabbit secondary antibodies (Novolink™ Polymer Detection System, Novocast). Immunoperoxidase staining was accomplished with diaminobenzidine tetrahydrochloride (DAB) and hematoxylin QS. The stained samples were imaged using a DMI300 inverted bright field microscope (Leica, UK) with a 40x objective lens.

### Cell Counting

The number of cells within printed constructs was determined by either manual cell counting of a single-plane image using the “Cell Counter” plug-in for Fiji, or by automated cell counting of a z-stack image employing the “3D Object Counter” plug-in for Fiji (Supplementary Methods). The value determined by automated object counting was referred to as the unmodified count with the number of cells assumed to be the number of objects counted. The resolved count assumed that the number of cells per automated counted object was equal to the object’s volume divided by the mean cell volume. Cell counting was performed on constructs stained with CAM, PI, DAPI and phospho-histone H3 antibodies.

### Quantification of SOX-9 mRNA expression

Printed oMSC constructs and oMSC pellets (using oMSCs from four different sheep) were cultured in medium with or without TGF-β3 over 3 to 10 d (Supplementary Methods). Lysis of oMSCs was performed by using a standard RNA extraction kit (RNeasy Mini Kit, QIAGEN, Supplementary Methods). The RNA concentration was determined with a spectrophotometer (Beckman Coulter DU530, Life Science) before cDNA synthesis with a PrimeScript RT Reagent Kit (Takara) and a thermal cycler (MJ Mini personal thermal cycler, Bio-Rad, Supplementary Methods). Absolute quantification of gene expression was performed with a QuantStudio^™^ 3D Digital PCR 20k Chip Kit (Thermo Scientific, Supplementary Methods). Briefly, samples were prepared by mixing 5 µL cDNA in RNase-free water with 7.3 µL QuantStudio^™^ 3D Digital PCR Master Mix, 1.5 µL RNase free water (QIAGEN) and 0.7 µL of primer solution (Taqman^®^ Gene Expression Assay, SOX-9 Hs01001343_g1 and β-Actin Hs01060665). The cDNA was amplified in the dPCR chip with a thermal cycler (Applied Biosystems^®^ Proflex PCR System^™^, Life Technologies) and then analysed by using a QuantStudio^™^ 3D Chip Reader.

### Statistics

The viability of HEK-293T constructs (*n* = 5) and oMSC constructs (*n* = 5) are given with standard deviation errors in the text and were calculated as the arithmetic means. Normalisation of SOX-9 mRNA expression in printed constructs (*n* = 22) and pellets (*n* = 24) as recorded by dPCR (Supplementary Fig. 20), was calculated for each culture condition as the mean expression of SOX-9 mRNA divided by the mean β-actin mRNA expression. The constructs or pellets analysed for each culture condition, comprised of the same four oMSC sets (derived from separate sheep), with each set of oMSC assays repeated 2 or 3 times. The significance of normalised SOX-9 mRNA expression between the culture conditions, was analysed by a paired *t*-test (Fig. 4), with two-tailed *p* values < 0.05 considered significant.

## Electronic supplementary material

Supplementary Information
