# Supplementary material for: High-Resolution Patterned Cellular Constructs by Droplet-Based 3D Printing
Source: Sci Rep. 2017 Aug 1;7:7004. doi: 10.1038/s41598-017-06358-x (PMC5539110; doi:10.1038/s41598-017-06358-x)
Supplement: Supplementary file 1 — Supplementary Information [file 41598_2017_6358_MOESM1_ESM.doc]

*submitted to Scientific Reports*

**Title**

**High-Resolution Patterned Cellular Constructs by Droplet-Based 3D Printing**

**Authors**

Alexander D. Graham**^†^**^1^, Sam N. Olof**^†^**^1^, Madeline J. Burke^2,3,4^, James P. K. Armstrong^2,3,4^, Ellina A. Mikhailova^1^, James G. Nicholson^5^, Stuart J. Box^1^, Francis G. Szele^5^, Adam W. Perriman^2^ & Hagan Bayley*****^1^

**Affiliations**

^1^Department of Chemistry, University of Oxford, Oxford OX1 3TA, UK. ^2^School of Cellular and Molecular Medicine, University of Bristol, Bristol, BS8 1TD, UK. ^3^Bristol Centre for Functional Nanomaterials, University of Bristol, Bristol, BS8 1TL, UK. ^4^Centre for Organized Matter Chemistry and Centre for Protolife Research, School of Chemistry, University of Bristol, BS8 1TS, UK ^5^Department of Physiology, Anatomy and Genetics, University of Oxford, Oxford, OX1 3QX, UK. **^†^**These authors contributed equally to the work. *****email: hagan.bayley@chem.ox.ac.uk.

**Supplementary Information**

**Supplementary Methods**

**S1. Cells & Culture Media**

**Cell Sources.** HEK-293/YFP and HEK-293/CFP were purchased from Cell Biolabs. Ovine mesenchymal stem cells (oMSCs) were extracted from the bone marrow of sheep at the Royal Veterinary College London, and were used to derive ovine osteoblasts at the University of Bristol. Murine chondrocytes were derived from murine mesenchymal stem cells that were isolated from mouse femurs by Dr Shang Zhang at the University of Bristol.

The HEK-293T derivative was a gift from the Schofield research group, University of Oxford. The cell-line was analysed using the Powerplex 16 HS short tandem repeat profiling (STR) system. The resulting profile was indicative of either genetic instability or cell-line contamination (Supplementary Cell Profile 1 and 2). Even though we appreciate there is an increasing awareness of cell-line contamination, which is an issue for life science research, in this instance the HEK-293T derivative was tested with our printing technique to probe cell properties such as viability and proliferation. Thus, the HEK-293T derivative was used as a valid generic cell model, rather than for its embryonic kidney properties. Additionally, the HEK-293T derivative tested negative for mycoplasma, using a MycoAlert™ Mycoplasma Detection Kit (Lonza), as performed by representatives of the tissue culture facility of the University of Oxford.

**Culture Medium for 2D Cell Cultures.** All cell lines were cultured in a minimal essential medium (MEM) with or without the following supplements: 10% v/v foetal bovine serum (FBS, Sigma-Aldrich), 2 mM GlutaMAX™ (Life Technologies), 0.1 mM MEM non-essential amino acids (Sigma-Aldrich), 10 to 25 mM HEPES (pH 7.0 to 7.6, Sigma-Aldrich) and 100 μg mL^-1^ penicillin and 100 μg mL^-1^ streptomycin (Life Technologies). The culture medium used with fluorescent protein expressing HEK-293 cells was supplemented immediately prior to use with 10 μg mL^-1^ blasticidin (Life Technologies). Ovine mesenchymal stem cells (oMSC) were grown in medium freshly supplemented with 5 ng mL^-1^ recombinant human fibroblast growth factor (PeproTech).

**Culture Medium for Printed HEK-293T Cells.** The culture medium used for printed HEK-293T cellular constructs was Dulbecco’s Modified Eagle’s medium (DMEM ,D5564, Sigma-Aldrich) supplemented with 10% v/v FBS, 2 mM GlutaMAX™, 0.1 mM MEM non-essential amino acids, 10 to 25 mM HEPES (pH 7.0 to 7.6) and 100 μg mL^-1^ penicillin with 100 μg mL^-1^ streptomycin.

**Culture Medium for Printed oMSCs.** The culture medium used for the culture of oMSC containing constructs and oMSC pellets was DMEM D5671 (Sigma-Aldrich) supplemented with 10.0 mg mL^-1^ bovine insulin, 5.5 mg mL^-1^ human transferrin and 6.7 μg mL^-1^ sodium selenite diluted from 100× ITS solution (Life Technologies), 2 mM GlutaMAX™, 1 mM sodium pyruvate (Sigma-Aldrich), 10 to 20 mM HEPES (pH 7.0-7.6) and 100 U mL^-1^ penicillin and 100 μg mL^-1^ streptomycin. For chondrogenesis experiments, the culture medium was freshly supplemented with 100 nM dexamethasone (Sigma-Aldrich) and 80 μM ascorbic acid-2-phosphate (Sigma-Aldrich), with or without 10 ng mL^-1^ recombinant human transforming growth factor-β3 (TGF-β3, R&D Systems). On days 8 to 10 of chondrogenesis experiments, the culture medium was freshly supplemented with 10 ng mL^-1^ insulin (Sigma-Aldrich).

**S2 Common Oil Solutions**

**Oil Solutions.** Undecane, silicone oil AR20, hexadecane and mineral oil (M3516) were purchased from Sigma-Aldrich*.* Prior to use*,* oils were mixed at the desired volume ratios and filtered through a 0.22 μm polyethersulfone membrane (Merck Millipore). The initial print oil composition (Supplementary Table 1) was used for preliminary print optimisation experiments (Supplementary Figure 2, Supplementary Figure 3, Supplementary Figure 4, Supplementary Figure 5 and Supplementary Figure 7). For all other prints, an optimised print oil for bioinks was used (Supplementary Table 1).

**Supplementary Table 1**: Commonly used oils involved in each of the steps of the print process

| **Standard Oil Solutions** | **Bulk Solvent** | **Lipid Content** | **[Lipid]**  **/ mM** |
| --- | --- | --- | --- |
| initial print oil | 50:50 v:v hexadecane: silicone oil AR20 | DPhPC | 1.2-1.5 |
| standard print oil for bioinks | 35:65 v:v undecane: silicone oil AR20 | DPhPC | 1.2-1.5 |
| oil for dilution, pre-phase-transfer | silicone oil AR20 | none | N/A |
| two-phase column oil (for phase transfer) | 75:25 v:v hexadecane: mineral oil (M3516) | none | N/A |

**S3. Bioink Stock Solutions**

**Agarose Solutions.** Ultra-low gelling temperature (ULGT) agarose powder (A5030, Sigma Aldrich) was used to make a 13 to 15 mg mL^-1^ ULGT-agarose solution. Warmed ULGT-agarose powder (typically ~12 mg) was dissolved in warmed culture medium (typically ~0.8 mL) at 65°C. To aid dissolution, the solution was vortex mixed and mechanically perturbed by pipette aspiration or sonication (40 kHz). The ULGT-agarose solution was kept warm in a water bath (65°C). The base culture medium used was Opti-MEM® (31985-062, Life Technologies) for HEK-293 cell lines, or serum-free DMEM with supplements (see Supplementary Methods S3) for oMSC, osteoblasts and chondrocytes.

**Fmoc-Dipeptide Hydrogel Stock Solutions.** Fluorenylmethyloxycarbonyl (Fmoc)-dipeptide gelators were freshly prepared as 10 mM Fmoc-isoleucine-glycine (Fmoc-IG) and 10 mM Fmoc-phenylalanine-phenylalanine (Fmoc-FF) stock solutions. The solutions were used within a week and pH corrected, if necessary, before use. Powder aliquots of Fmoc-dipeptides (Bachem) were stored at ‑20°C and used to prepare the stock solutions. Fmoc-IG (16 mg) and Fmoc-FF (12 mg) were separately dissolved in Milli-Q® water (1.5 mL) with 1 M sodium hydroxide (10 to 20 μL), and left to stir at room temperature (10 to 15 mins). The partially solubilised Fmoc-dipeptide solution was sonicated (20 min, 37°C, 40 kHz), pH corrected with 0.1 mM sodium hydroxide to either 8.50 (Fmoc-IG) or 10.50 (Fmoc-FF) and then diluted to 3 mL total volume in Milli-Q® water.

**10 mM Fmoc-XX Solution.** The Fmoc-XX solution was an equimolar ratio solution of Fmoc-IG and Fmoc-FF. For a 10 mM Fmoc-XX solution, 10 mM Fmoc-FF and 10 mM Fmoc-IG were mixed at a 1:1 (v:v) ratio and then sonicated (40 kHz, 5 min). Fmoc-XX was made fresh on the day of use.

**Collagen Protein Supplement**. Type I collagen was prepared by diluting concentrated stock solutions into working solutions. The stock solution was 5.0 mg mL^-1^ bovine type I collagen (Life Technologies) and was stored as aliquots at 4°C. The type I collagen working solution (3.0 mg mL^-1^) was prepared by mixing ice cold reagents in the order: type I collagen stock (50 μL), 10× concentrate of Dulbecco’s phosphate-buffered saline (10× PBS, 8.3 μL, Sigma-Aldrich), 1 N sodium hydroxide (1.3 μL) and Milli-Q® water (23.8 μL). The collagen working solution was prepared immediately prior to its addition to the bioink in a laminar flow biological safety cabinet.

**Bioink Preparation**. Prior to cell addition the bioink consisted of an 8:1 v:v mixture of ULGT-agarose solution to Fmoc-dipeptide solution, with or without collagen. In typical experiments, 13 to 15 mg mL^-1^ ULGT agarose solution was heated to 65°C. To this, 10 mM Fmoc-XX was added. All solutions were maintained above the agarose gel melting temperature (*i.e.* ~50°C) until the addition of collagen or cells, at which point the solution was cooled and kept at 37°C. Where collagen was used, the bioink comprised a 9:1 v:v mixture of the agarose with Fmoc-XX solution to phosphate buffer saline (PBS) which contained diluted type I collagen working solution. Collagen was typically supplemented at a final concentration of 15 μg mL^-1^ to the bioink, which was sonicated (5 min, 37°C, 40 kHz) immediately prior to cell addition in a Branson 2800 ultrasonic bath. For sterility, solutions were irradiated with UV (15 min, 365 nm) prior to the addition of cells. Solutions were placed 4.5 cm beneath an UV LED (Eclipse-M365L2-C5, Nikon) controlled by an LED driver (LEDD1B, Thorlabs) set to half power.

In typical experiments involving HEK-293T (a non-adherent cell-line), confluent cells from a T25 culture flask were resuspended in Opti-MEM® (5 mL), and an aliquot (typically 1 to 2 mL) was pelleted by centrifugation (3 to 5 min, 300 to 500 × g). The pellet was resuspended in the bioink at the required cell density. For adherent cell lines, such as HEK-293/YFP and oMSCs, the culture was trypsinised and centrifuged (3 to 5 min, 300 to 500 × g), and the pellet resuspended in culture medium. Cell resuspension was typically in 100 to 200 μL of bioink at 5 × 10^6^ to 15 × 10^6^ cells mL^‑1^, most often 15 × 10^6^ cells mL^-1^. Cell densities of harvested cells were measured with an automated cell counter (Life Technologies, Invitrogen Countess II FL). The counter imaged cells at 5-megapixel resolution in the bright-field channel at 2.5× optical magnification and analysed the image using advanced counting algorithms. All bioink solutions were used on the day they were made.

**S4. 3D Printing**

**The 3D Printer**. The droplet-based 3D printer used for all bioprinting experiments was described in a previous publication^1^. In brief, the printer consisted of a static piezoelectric actuated droplet generator, which ejected droplets from its oil-submerged nozzle into a lipid-in-oil bath. The bath rested on a motorised stage (PatchStar micromanipulator, Scientifica), which moved along three orthogonal axes in *x* (horizontal plane), *y* (horizontal plane) and *z* (vertical plane) dimensions. Printing was monitored by a side-on stereomicroscope (SMZ660, Nikon), one ocular of which held a web-camera (Lifecam HD-3000, Microsoft) to record videos and images of printing. Droplet ejection was triggered by the application of a square-wave voltage pulse to the piezo disc of the droplet generator. In-house print programs allowed both user triggered droplet ejection and automated printing of aqueous constructs based on digital “maps”. During automated printing, the movement of the stage was synchronised with droplet ejection.

For the work presented here, various components of the previously described 3D printer were modified to improve printing and component longevity. Firstly, the oil container was changed to a truncated glass cuvette (3.1125/SOG/10, Starna), which was held in place by a machined polymethyl methacrylate (PMMA) support attached to the motorised manipulator. To improve the longevity of the droplet generator, the plastic housing used to hold the piezo-electric disk, was widened compared to the original design^1^ such that the piezo was less exposed. A new piezo driver was built with a voltage output of ±33 V by the electronic workshop of the Chemistry Department at the University of Oxford . Thus, a voltage pulse with peak-to-peak amplitude of between 0 and 66 V could be applied the piezo-electric disk. Finally, the microscope was mounted using a custom microscope gantry engineered in-house (mechanical workshop, Chemistry Department at the University of Oxford).

**Portable Printer**:  A portable version of the printer was fabricated and used for select prints. These prints included the dual nozzle printing of the junction with a diagonal plane cellular interface, and the single nozzle printing of oMSC constructs cultured with TGF-β3 for 35 days.

The portable printer was constructed by placing the original printer hardware, except for the imaging components, into a bespoke flight-case (580 × 430 × 430 mm^3^, The Flightcase Company). Due to size considerations, the original imaging components were replaced with a 1280 x 1024 pixel USB camera (DCC1645C, Thorlabs), and a variable magnification lens system comprised of a zoom lens (MVL6X12Z, Thorlabs), extension tube (MVL05A, Thorlabs) and mounting adapter (MVLCMC, Thorlabs). This system had a resulting field of view across the diagonal of the image in the range 2.67 to 17.16 mm, depending on magnification setting.

The portable printer was controlled by a laptop using custom software written in LabVIEW (National Instruments). This new software consolidated the manual and map-guided functionalities of the previous Processing programs^1^ into a single user interface. It also allowed communication with the camera of the portable printer, such that it displayed a live image-feed for visualisation, and could save either images or video to disk.

**3D Printer Sterilisation.** The droplet generator's aqueous chamber and rubber capillary holder, as well as the glass cuvette (used to hold oil and act as a substrate), were all thoroughly cleaned with Milli-Q® water and ethanol before printing. They were then dried under a nitrogen stream. On the day of use, the print nozzles (*i.e.* customised glass capillaries) were flushed with Milli-Q® water, ethanol, and isopropanol, and were then dried under a nitrogen stream. The cleaned capillaries were subsequently treated with oxygen plasma (8 min, 5 to 10 SCCM) in a plasma cleaner (Femto version A, Diener Electronic). Finally, the droplet generator was filled with Milli-Q® water and a capillary was inserted.

**3D Printing**. The bioink was gently vortex mixed (1 to 2 s), placed in a well-loading array (10 μL) alongside hexadecane (7 μL) and suction loaded (~4 μL) into the print nozzle beneath a hexadecane oil plug (~1 to 2 μL). The outside of the tip of the glass nozzle was subsequently cleaned with lens tissue soaked in Milli-Q® water. The nozzle was submerged into the print oil (usually 200 μL) and the droplet generator was continuously fired with square wave pulses of varying voltage and duration until conditions were found for the reproducible ejection of singlet droplets of uniform size. For bioinks, typical square wave pulse parameters were 50 to 350 μs pulse-width with peak-to-peak voltage amplitudes of 40 to 63 V. The diameter of ejected droplets was typically ~130 μm, but between experiments varied over the range 120 to 160 μm. In parallel with droplet tuning, bioink gelation was tested by an inverted vial test. If the bioink gelled after cooling (4°C, 20 min) automated printing was conducted. In practice, the applied voltage of the print pulse was gradually increased throughout a print session of multiple aqueous constructs to maintain consistent droplet production. Generally, multiple constructs were printed successively within the same print chamber, with the last construct left to stand for 5 min before moving.

**Preliminary Bioink-Based Printed Construct**. A cell-free ink was printed as 9 × 11 droplets for 5 layers to display the packing within a printed construct (Fig. 1b). The ink comprises 13 mg mL^-1^ ULGT-agarose in Opti-MEM® with 5 μM sulforhodamine 101 (Life Technologies). The preliminary HEK-293T cellular construct shown in Fig. 1e was printed as 11 × 14 droplets for 2 layers in a 50:50 v:v mixture of hexadecane to silicone oil AR20 containing 1.2 mM DPhPC. Here the bioink comprises 1.0 mM Fmoc-XX, with 5 μM calcein‑AM and 5 μM propidium iodide, in 70% v/v culture medium and 30% v/v Milli-Q® water.

**Constructs with a Single Cell Population**. Constructs of a single cell-type

were printed as layers of droplet sheets (2 to 4) with a range of horizontal droplet dimensions from 7 x 8 to 11 x 14. For phase transfer experiments, 4 layers was the usual thickness.

**Constructs with Two Cell Populations.** Two cell type constructs were printed using two different methods. Either a single nozzle/ droplet generator was used to sequentially print discrete bioinks or two nozzles/ droplet generators were used in tandem with discrete bioinks. The single nozzle approach was used for the lamellar, Y-shaped and cruciform patterned constructs. Here, cell-type 1 was printed and then, after nozzle cleaning, cell-type 2 was printed. The nozzle was cleaned by sequential immersion of the tip in the following solvents: Virkon® disinfectant, 8 M sodium hydroxide, and then Milli-Q® water at 65°C. Additionally, before loading the second bioink into the nozzle, the aqueous reservoir of the droplet generator was replenished with water.

All lamellar constructs were fabricated as follows: initially a wider lower droplet sheet (typically 7 × 9 droplets) was printed for 3 to 4 layers followed by perimeter-only droplets for a further two layers to correct for droplet rolling at the edge of the construct. A narrower upper sheet (typically 6 × 7 droplets) was then printed directly on top of the lower sheet for 3 layers. The lamellar constructs studied over 5 d of culture also had perimeter-only droplets (6 × 7 droplet dimensions) printed on the upper droplet sheets for two layers.

The Y-shape and cross patterned constructs were printed as follows. The Y-shape and cross patterns were first printed for 3 to 4 layers, followed by the flanking droplets, surrounding the pattern for 2 to 6 layers. Finally, an additional 2 layers were printed on top of the construct. The outer dimensions were 10 × 12 and 8 × 9 droplets for the cruciform and the Y-shaped constructs, respectively.

The dual nozzle approach was used to print the cellular junction with a diagonal interface in the *x*-*z* (vertical) plane (Supplementary Figure 8). Here, two droplet generators were employed in tandem to print the two cell populations layer-by-layer. The junction was printed as 21 × 24 droplets for 7 layers.

**S5. Gel Encapsulation and Phase Transfer of Cell Constructs**

**Gelation of Printed Cellular Constructs (Under Oil).** The printed constructs were gelled after printing by cooling from room temperature and standing at 4°C for approximately 20 to 25 min. With the internal contents of the printed constructs gelled, the constructs were gel encapsulated and transferred from oil to culture medium under sterile conditions as described. First, the print oil surrounding the gelled constructs was reduced to ~100 to 150 μL at ambient temperature. Silicone oil AR20 (200 μL) was gently added to the corner of the cuvette and mixed oil (200 μL) was then removed from the diagonally opposite corner. The silicone oil addition, and mixing and removal step was repeated three more times. 13 to 15 mg mL^-1^ ULGT-agarose previously heated to 50°C (0.2 to 0.4 μL) was then pipetted onto each construct. If the agarose drop did not coat the printed construct, a silver wire (⌀ 0.1 mm, Sigma-Aldrich) was used to manipulate the droplet onto the construct. The coated printed constructs were then gelled (4°C, 20 to 25 min). Coated constructs were either phase transferred immediately or stored in a hydrated environment within a cell incubator (37°C, 5% CO_2_).

**Phase Transfer of Printed Cellular Constructs**. Phase transfer was performed by adapting a protocol described by Ces and co-workers^2^. Oil-above-culture-medium interfaces were prepared in the wells of a sterile 8-well microscope slide (II Chamber Slide™, Lab-Tek™). The lower aqueous phase was ~300 μL of culture medium, specific to the cell-line (Supplementary Methods S1). The upper oil phase was ~200 μL of a 3:1 v:v mixture of hexadecane to mineral oil. The two-phase columns were placed for ≥15 min in a cell incubator (37°C, 5% CO_2_). Printed constructs were transferred from the oil-containing print chamber by pipette into the upper oil phase and allowed to fall through the interface. After transfer, the oil phase was discarded and 300 μL of additional culture medium was added. The chamber was then placed inside a cell incubator (37°C, 5% CO_2_) for long term culture.

**S6. Maintenance of Cellular Constructs**

**Culture of Printed Cellular Constructs**. The phase-transferred printed constructs were cultured for up to 10 d in a cell incubator (37°C, 5% CO_2_) within wells of a microscope slide (II Chamber Slide™, Lab-Tek™) containing culture medium (~600 μL per well). Every 2 to 3 d the culture medium was changed: ~200 to 300 μL of culture medium was carefully removed near the air-culture-medium interface and 300 μL fresh culture medium was added slowly at corner of the well, a process that was performed 2 to 3 times each medium change. The culture medium was added at low flow rate using a 100 μL micropipette to avoid disrupting the printed constructs.

**S7. Imaging of Printed Cellular Constructs**

**Imaging of the Constructs.** Printed cellular constructs were imaged under culture medium within the wells of sterile microscope slides (II Chamber Slide™, Lab-Tek™). Constructs were viewed by either a widefield fluorescence microscope (Leica DMI 8) or confocal fluorescence microscopes (Leica SP5 or Zeiss LSM 710), in both bright field and fluorescence modes.

**Confocal Microscopy**. Printed cellular constructs were imaged using a confocal microscope (SP5, Leica) with a HC PL FLUOTAR 10× objective lens (0.3 N.A.), in both transmitted light and fluorescence modes. To improve contrast, the transmitted light mode used a scanning polarising differential interference contrast (DIC) setting. Both 2D images and *z*-stack optical sections were recorded of each printed construct, using image resolutions of 512 × 512, 1024 × 1024, 2048 × 2048 or 4096 × 4096 pixels and frame averaging of 1 to 6 frames. Optical sections of printed constructs were recorded using spacing of 2 to 10 μm between each slice, commonly 5 μm. For all fluorescent samples including fluorescently dyed cell constructs, the PMT emission-band and laser wavelength were kept constant for a specific dye. However, the laser power and gain varied from sample to sample to compensate for the effective concentration of the dye at the time of imaging. The confocal pinhole was set to 70.78 for imaging of all samples. Supplementary Table 2 summarises the imaging settings used for all commonly used fluorescent dyes.

**Supplementary Table 2**: Settings for confocal microscopy (Leica SP5) of fluorescent samples. Samples were stained with a fluorescent dye at the concentration indicated. The fluorophores excitation and emission wavelength maxima are also stated. The laser wavelength and emission range detected by the PMT were kept constant between samples for a given fluorophore. The laser power and PMT gain settings employed varied from sample to sample and the typical values employed has been given.

| **Fluorescent Dye** | **[Dye] / µM** | **λ Ex_max_**  **/ nm** | **λ Em_max_**  **/ nm** | **λLaser**  **/ nm** | **PMT range**  **/ nm** | **Laser power / %** | **PMT gain**  **/ V** |
| --- | --- | --- | --- | --- | --- | --- | --- |
|
| **sulforhodamine 101** | 5 | 586 | 605 | 543 | 570 to 665 | 35 | 900 |
| **POPO™-3 iodide** | 1 | 534 | 570 | 543 | 600 to 650 | 50 | 1100 |
| **calcein-AM** | 5 | 490 | 515 | 488 | 500 to 550 | 10 | 500 |
| **propidium iodide** | 5 | 535 | 615 | 543 | 580 to 660 | 20 | 900 |
| **HEK-293/YFP** | N/A | 514 | 527 | 514 | 525 to 575 | 30 | 550 |
| **Red CMPTX CellTracker™** | 5 | 577 | 602 | 633 | 645 to 700 | 30 | 750 |
| **Deep Red CellTracker™** | 1 | 630 | 660 | 543 | 550 to 615 | 40 | 1000 |

**Live/Dead Cell Staining.** Live/dead staining of cellular constructs was performed using a calcein-AM (CAM) dye (Cambridge Biosciences Ltd) and a propidium iodide (PI) dye (Sigma Aldrich). Stock solutions of 5 mM PI in DMSO and 5 mM CAM in Milli-Q® water were prepared by dissolution of the dyes. A working solution containing 0.05 mM of each dye was freshly prepared by pipetting 2 μL of each stock solution into 196 μL of culture medium. A portion of the working solution was either added directly to the cells prior to printing, or to the medium surrounding printed constructs at a final individual dye concentration of ~5 μM. After staining and imaging, cellular constructs for immunocytochemistry were fixed.

**CellTracker Staining**. CellTracker™ dyes (Life-Technologies), Red CMPTX (RC) and Deep Red (DR) were used to fluorescently stain cells to visualise their printed patterns. Stock solutions of CellTracker™ were supplied as 10 mM RC in DMSO and 1 mM DR in DMSO. Both stocks were diluted into serum-free culture medium to give working dye solutions: 5 μM RC (0.05% v/v DMSO) and 1 μM DR (0.1% v/v DMSO). Staining was typically performed as follows. A flask of confluent cells was washed with PBS, with care taken not to delaminate the cells, and then one of the dye solution was added (2 mL for a T25 culture flask). The flask was placed in a cell incubator (37°C, CO_2_) for 15 to 45 min. After incubation, the cells were suspended in serum-free culture medium and centrifuged (400 × g, 4 min). The pellet was washed with PBS and resuspended in the bioink. For pattern visualisation experiments, osteoblasts and HEK-293T cells were stained with CellTracker™ RC, whilst chondrocytes, HEK-293/YFP and HEK-293T were stained with CellTracker™ DR.

**Immunocytochemistry.** Immunocytochemistry (ICC) of the printed constructs was performed within the microscope chamber slides. Printed constructs were first fixed in 4% v/v paraformaldehyde (Sigma-Aldrich) and then quenched with 50 mM glycine (Sigma-Aldrich). Fixed constructs were gently washed in PBS, and then blocked for 1 h at room temperature with PBS supplemented with 10% v/v donkey serum (Bio-Rad) & 0.1% v/v Triton-X 100 (Fisher Scientific). Primary antibodies in the blocking solution were added to the printed constructs which were then incubated for 2 h at 37°C. After washing in PBS, the constructs were incubated (2 h, 37°C) in blocking solution containing Alexa conjugated secondary antibodies. Finally, the constructs were washed in PBS, incubated at room temperature (15 min) in PBS containing 2.9 μM DAPI (Sigma-Aldrich) and washed again.

The primary antibodies were: 0.25% v/v rabbit anti-phospho-histone 3 (Merck Millipore: 06-570)^3^ and 0.67% v/v rabbit anti-SOX-9 (Merck Millipore: AB5535). Secondary donkey antibodies conjugated to Alexa Fluor 568 (Invitrogen: A10042) or 647 (Invitrogen: A-31573) were used with the SOX-9 and phospho-histone 3 antibodies, respectively. Aberrant secondary antibody staining was checked by omitting the primary antibody and was not observed. SOX-9 staining was optimised on oMSCs bound within gelled bioink blocks (~1 μL) after 7 d culture. The blocks comprised of standard bioink solution supplemented with 15 μg mL^-1^ type I collagen and oMSCs at 15 × 10^6^ cells mL^‑1^. All ICC samples were imaged using a Zeiss, LSM 710 confocal microscope.

**Immunohistochemistry of oMSC samples**. Immunohistochemistry was performed on sections of oMSCs samples after 35 d of culture with TGF-β3 (Supplementary Methods). Samples were rehydrated by sequential immersions of 2 min duration in xylene, 100%, 90%, 80% and 70% (v/v) ethanol and deionized water. Immunohistochemistry was performed on sections with rabbit anti-type I collagen (Abcam: AB34710) and rabbit anti-type II collagen (Abcam: AB34712) primary antibodies, and polymeric HRP-conjugated anti-rabbit secondary antibodies (Novolink™ Polymer Detection System, Novocast). Immunoperoxidase staining was accomplished with diaminobenzidine tetrahydrochloride (DAB) and hematoxylin QS. See the sections immediately below for further details regarding the antigen retrieval, antibody blocking and staining, and IHC stock solution preparation. The stained samples were imaged using a DMI300 inverted bright field microscope (Leica, UK) with a 40× objective lens. IHC images (Figure 4e and Supplementary Figure 1a-i) were white-balanced using a Fiji plugin, white balance correction.ijm^4^ (developed by Vytas Bindokas, 2006 and modified by Patrice Mascalchi, 2014).

**Antigen Retrieval** **Procedure (IHC)**. Prior to immunohistochemical staining, enzymatic antigen retrieval was performed on both oMSC pellet and printed oMSC samples. Two solutions were prepared for enzymatic antigen retrieval; 10 mg mL^-1^ hyaluronidase (Sigma Aldrich) in PBS and 2 mg mL^-1^ pronase (Roche, Switzerland) solution in PBS. Hyaluronidase solution (200 μL) was added to each section and the slides were incubated in a humidified chamber at 37°C for 30 min. The enzyme solution was removed by immersing the slide in PBS for five minutes. Pronase solution (200 μL) was then added to each section, and the slides were incubated in a humidified chamber at 37°C for 30 min, followed by removal through immersion in a solution of PBS. 3% v/v hydrogen peroxidase solution (200 μL) was added to each section and the slide was incubated in a humidified chamber at room temperature for five min. The enzyme solution was removed by immersing in a solution of PBS for 5 min. A solution of Tween in tris-buffered saline (TBS) was prepared by dissolving 13.15 g of sodium chloride, 9.08 g Tris, and 0.75 mL Tween®-20 in 1.5 L of deionised water (pH 7.5-7.6).

**Blocking and Antibody Staining (IHC)**. Blocking solution (30 mg mL^-1^ bovine serum albumin (BSA) in Tween and TBS solution, 200 μL) was added to each section, and the slides were incubated in a humidified chamber at room temperature for 1 h. The blocking solution was then removed with three 10 min immersions in PBS.

**DAB and Hematoxylin Staining Procedure (IHC)**. Primary antibody solution (70 μL) or isotype control was added to each section and incubated overnight in a humidified chamber at 4°C. Secondary antibody solution was then introduced (70 μL), incubated (1 h) and subsequently removed by tipping excess solution from the slide. Diaminobenzidine tetrahydrochloride (DAB) chromogen substrate (Novocast) solution (200 μL) was added to the sections and incubated (10 min). The excess DAB solution was removed by tipping away excess solution and rinsing with deionised water. Finally, a few drops of hematoxylin QS nuclear counterstain was added directly to slides and incubated from 1 min.

**Working Solutions for IHC**. Primary antibody solutions were prepared in antibody diluent (comprising of 10 mg mL^-1^ BSA in Tween and TBS solution). In addition, a 1% v/v rabbit anti-type I collagen (Abcam: AB34710) and 5% v/v rabbit anti-type II collagen (Abcam: AB34712), as well as an isotype control of 5% v/v goat IgG Isotype (Santa Cruz, USA) were prepared in an antibody diluent. Secondary antibody working solutions comprised of 6 μL polymeric HRP-conjugated anti-rabbit secondary antibody stock (Novolink™ Polymer Detection System, Novocast) diluted in 1.2 mL of antibody diluent. Diaminobenzidine tetrahydrochloride (DAB) chromogen substrate was prepared by adding 4 drops of DAB chromagen (1.74% w/v 3,3’ – diaminobenzidine, in a stabiliser solution, Novocast) to DAB diluent substrate buffer (4 mL, Novocast). Hematoxylin nuclear counterstain (<0.1% Hematoxylin, Novocast) was added directly to slides without any additional preparation steps.

**S8. Characterisation of Printed Cellular Constructs**

**Image Processing.** Images were processed and rendered with the software Fiji^5,6^ (<http://fiji.sc/Fiji>). 3D projections, referred to as 3D reconstructions, of printed constructs were formed from *z*-stack image series using the 3D project plug-in for FiJi^6,7^. The plug-in created a 3D object that was rotated from 0 to 360° and the 0° frame was selected as the image to represent the 3D projection. The projection method selected was “brightest point” and the image series was interpolated, a function which uses z-scaling to eliminate gaps between the image slices.

**Cell Counting of Cellular Constructs.** The number of cells within printed constructs was determined by either manual cell counting using the “Cell Counter^8^” plug-in for Fiji, or automated cell counting employing the “3D Object Counter^9,10^” plug-in for Fiji. Typically, manual cell counting was performed on one image of a *z*-stack of the printed construct, commonly at 30 to 60 μm height i.e. within the lowest droplet layer. The cell occupancy of droplets in constructs printed with bioinks containing different cell densities was performed manually, in this case the occupancy was determined for 25 droplets.

Automatic object counting was used to determine numbers of cells which were stained with either CAM, PI, PH3 antibodies or DAPI. Here, a z-stack of the entire cell construct in culture medium was split into individual channels (at 8-bit resolution) and a single fluorescence channel was subjected to automatic counting. Counting was performed with values selected for the intensity limit threshold and size filter range (i.e. minimum and maximum number of object voxels), and by selecting whether objects on the image edge were counted (Supplementary Table 3)^10^. Counting was repeated with varied threshold values and the value which counted the majority of cells was selected. These results were referred to as the unmodified count, but the method generally underestimated the number of live cell numbers as overlapping live cells were counted as a single object.

**Supplementary Table 3**: Settings of the 3D object counter plug-in used for automatic cell counting of printed constructs

| **Cellular Construct** | **Time-point / days** | **Dye** | **Minimum Voxel size / pixel^2^** | **Maximum Voxel size / pixel^2^** | **Edge Objects Counted** |
| --- | --- | --- | --- | --- | --- |
|
| HEK-293T | 0 | CAM | 15 | 8000 | No |
| HEK-293T | 0 | PI | 15 | 8000 | No |
| oMSCs | 0 | CAM | 15 | 8000 | No |
| oMSCs | 0 | PI | 15 | 8000 | No |
| HEK-293T | 3, 7 | DAPI | 80 | none | Yes |
| HEK-293T | 3, 7 | PH3 | 80 | none | Yes |
| HEK-293T | 3, 7 | PI | 80 | 8000 | Yes |
| oMSC | 3, 7, 10 | CAM | 40 | none | Yes |
| oMSC | 3, 7, 10 | PI | 40 | 8000 | Yes |

A more accurate value for cell numbers was determined by resolving the automated object count. Here, the volume of each counted object (*V*_object_ in voxels) was divided by the average cell volume (
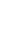
 in voxels) and the resulting quotient was then rounded to the nearest whole number. The summation of these quotients gave the total number of stained cells within the construct (Supplementary Equation 1) and was referred to as the resolved count. The average size for cells was determined for each digital magnification of z-stacks used by averaging the volume (in voxels) of single cells (*n* = 6 or 12). However, no resolution of the PH3 object count was performed because the cells were typically discrete across the construct. Consequently, each counted PH3 object was treated as one cell.

**Supplementary Equation 1**


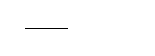


**Measurements of Features of Printed Constructs**. Measurements of features of printed constructs including layer thicknesses, width of patterned features and outer dimensions was performed on *z*-stack confocal micrographs of the constructs using Fiji. For the Y-shaped and cruciform patterned constructs, the width of the internal features was averaged over 12 measurements of a single construct. For the lamellar constructs, the thickness of the cell layers was measured as the vertical distance between frames of z-stacks. Specifically, between frames depicting when cells (in the lower layer) first appear and when they are no longer present in the centre of the printed construct. The thickness of the exterior gel coating of cell constructs was averaged from measurements of 3 constructs. These measurements were performed on the vertical edge of each construct 10 or 16 times.

**Viability of Printed Cells**. The viability of cells within constructs (Supplementary Table 4) was calculated as the proportion of live cells to the total number of printed cells, values of which were determined by cell counting.

**Supplementary Table 4**: Mean viabilities of printed cells immediately after print (day 0) and after culture (day 3, 7 and 10) as determined from automated cell count values. Standard error values are of the mean.

| **Printed Cells** | **Time in Culture / days** | **Mean Viability / %** | **Standard Deviation / %** | **Standard Error / %** | **Sample Size / constructs** |
| --- | --- | --- | --- | --- | --- |
| HEK-293T | 0 | 88.4 | 4.89 | 2.19 | 5 |
| HEK-293T | 3 | 96.8 | 1.3 | 0.7 | 4 |
| HEK-293T | 7 | 99.5 | 0.2 | 0.1 | 4 |
| oMSCs | 0 | 90.8 | 4.2 | 1.9 | 5 |
| oMSCs | 3 | 95.0 | 1.5 | 0.9 | 3 |
| oMSCs | 7 | 83.4 | 0.4 | 0.2 | 3 |
| oMSCs | 10 | 91.2 | 3.6 | 2.5 | 2 |

**Proportion of Mitotic Cells in Printed Constructs**. For printed HEK-293T constructs, the percentage of cells undergoing mitosis was determined by using PH3 and DAPI cell count values (Supplementary Equation 2).

**Supplementary Equation 2**


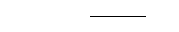


**Cell Density of Printed Droplets**. The average cell density of printed droplets (
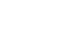
 in cells mL^‑1^) was determined from the average size of a droplet (
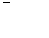
 in mL) and the average number of cells per droplet (
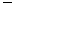
), see Supplementary Equation 3. The average diameter of the printed droplet was assumed to be equivalent to the spacing used between droplet ejections of printing, as this value was set such that droplets are just in contact as they print. A spherical volume was then calculated for this diameter and assigned a variance of ± 8% (as determined by the droplet uniformity of previous prints). The average number of cells per droplet was calculated from the manual cell counts of construct droplets (*n* = 25).

**Supplementary Equation 3**


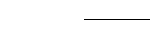


**Angle of the Sloping Cellular Interface.** Two cell populations were printed such that their interface formed a plane that was not parallel to any of the outside faces of the finished cuboidal construct (Supplementary Figure 12). To analyse the angle of this interface, confocal micrographs were taken at the planes *z*_1_=48 µm and *z*_2_=168 µm. Within each image at *z_i_*, the position, *p_i_*, of the cellular interface along the *x*-axis was measured at 12 different points along the *y*-axis and averaged (
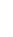
). Finally, the slope angle, *θ*, formed between the interface and substrate in the *x-z* plane was calculated using Supplementary Equation 4.

**Supplementary Equation 4**


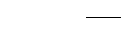


**Theoretical thickness of the exterior gel**. The thickness was calculated for a printed construct of 7 × 9 × 4 droplets, where each droplet was 150 μm in diameter, and the construct was coated in 0.2 to 0.4 μL of gel. The volume of the printed construct was calculated as the product of the droplet volume multiplied by the number of droplets (Supplementary Equation 5), which evaluates to 0.45 μL for the diameter and number of droplets given above.

The printed construct was modelled as a cuboid with sides of length *u*, *v*, and *w*, where *w* is the measurement normal to the substrate. The gel coating was assumed to spread evenly over all surfaces of the printed construct with a thickness, *t*, which resulted in a larger cuboid with sides of length (*u*+2*t*), (*v*+2*t*), and (*w*+2*t*). Therefore, the volume of the coated construct can be expressed as given in Supplementary Equation 6, and rearranged as given in Supplementary Equation 7.

Visual inspection of printed constructs revealed the ratio of side lengths was approximately 3:3:1, such that *u* = *v* = 3*w*. Under this assumption, the height of the construct, *w*, is given by Supplementary Equation 8. Substituting in values for *u*, *v*, and *w* (using the 3:3:1 ratio and Supplementary Equation 8), and for the coated construct volume (Supplementary Equation 9), allowed the solution of Supplementary Equation 7 for *t* using an online cubic equation solver^10^. The result was *t* = 45 or 81 µm for 0.2 or 0.4 μL of gel respectively.

**Supplementary Equation 5**


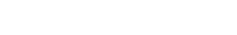


**Supplementary Equation 6**


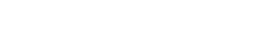


**Supplementary Equation 7**


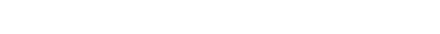


**Supplementary Equation 8**


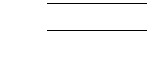


**Supplementary Equation 9**


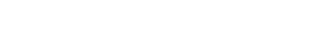


**S9. Differential Capacity of Printed oMSCs**

**Printed oMSC Constructs for Chondrogenesis**. A bioink composed of 15 × 10^6^ to 17 × 10^6^ oMSCs mL^-1^ was printed as cuboidal constructs (7 × 8 × 4 droplets), which were transferred to medium. The oMSC constructs were cultured with and without TGF-β3 (see Supplementary Methods S1 for full medium composition), and harvested at different time points (3, 7, 10 or 35 d). Printed oMSC constructs for digital polymerase chain reaction (dPCR) analysis were produced with oMSCs extracted from four different sheep: sheep 345 (*n* = 6), sheep 419 (*n* = 6), sheep 342 (*n* = 6), and sheep 394 (*n* = 4), with half the constructs cultured in the presence of TGF-β3 and the other half cultured in absence of TGF- β3

**Pellet Control Samples**. oMSCs were harvested and pelleted by centrifugation (1.2 × 10^6^ or 1.6 × 10^6^ cells). The pellets were resuspended in 1.5 or 2 mL of culture medium either with or without TGF-β3. The suspensions were aliquoted (0.5 mL, 0.4 × 10^6^ cells) into separate falcon tubes and centrifuged (500 × g, 5 min). Pellets under culture medium were incubated (37°C, 5% CO_2_) for 7 d or 35 d, with three medium exchanges a week. The cap of each tube was loosened to allow gas exchange. One pellet was cultured for each condition under study.

**oMSC Samples RNA Extraction and cDNA Synthesis**. Cell lysis was performed on printed oMSC constructs using a standard RNA extraction kit (RNeasy Mini Kit, QIAGEN). Culture medium (300 μL) was removed from every sample well and each construct was left to incubate (10 min) in 200 µL of lysis solution. The constructs were subsequently disrupted with a 20-gauge needle and then 200 µL of 70% v/v ethanol was added and mixed by pipette trituration. Each construct was then transferred to an individual RNeasy Mini spin column and centrifuged (8000 × g, 15 s). The column was twice washed with buffer before the RNA was eluted in RNase-free water and stored at -80°C. The RNA concentration was determined using a spectrophotometer (Beckman Coulter DU530, Life Science) before cDNA was synthesized using a PrimeScript RT Reagent Kit (Takara) and amplified with a thermal cycler (MJ Mini personal thermal cycler, Bio‑Rad). The input sample volume was 40 µL and the program employed was as follows: 37°C for 15 min, 85°C for 5 s and a 4°C cooling period. Extraction on pellets was performed in the same manner as for the printed oMSC constructs and then were analysed by dPCR. In this case, the initial extraction steps, lysis buffer addition and needle trituration were performed in the falcon tube.

**Printed oMSC dPCR Analysis**. Absolute quantification of gene expression was performed using a QuantStudio™ 3D Digital PCR 20k Chip Kit (Thermo Scientific). Samples were prepared by mixing 5 µL cDNA in RNase-free water with 7.3 µL QuantStudio™ 3D Digital PCR Master Mix, 1.5 µL RNase free water (QIAGEN) and 0.7 µL of primer solution (Taqman® Gene Expression Assay SOX-9 Hs01001343_g1 and β-Actin Hs01060665). The sample was then transferred to a dPCR chip, using a QuantStudio™ 3D Digital PCR Chip Loader, which was then covered in immersion fluid and sealed using UV glue. Amplification was performed using a thermal cycler (Applied Biosystems® Proflex PCR System™, Life Technologies) with the default sequence: 96°C for 10 min, 60°C for 2 min followed by 98°C for 30 s (for 39 ×), 60°C for 2 min and a 10°C cooling period. Finally, chips were removed from the thermal cycler, allowed to warm to room temperature and analysed using by a QuantStudio™ 3D Chip Reader. A no template control showed no signal expression of beta-actin or SOX-9.

**Sectioning of fixed oMSC samples.** Printed and pellet oMSC samples cultured over 35 days (Supplementary Methods S9) were fixed in 4% paraformaldehyde (Supplementary Methods S7). Fixed samples were embedded in HistoGel™ (Thermo Scientific) and processed by successive ethanol and xylene wash steps. Processed samples were embedded in paraffin wax and sectioned as 4 µm slices with a RM2255 rotatory microtome (Leica).

**Supplementary Figures**


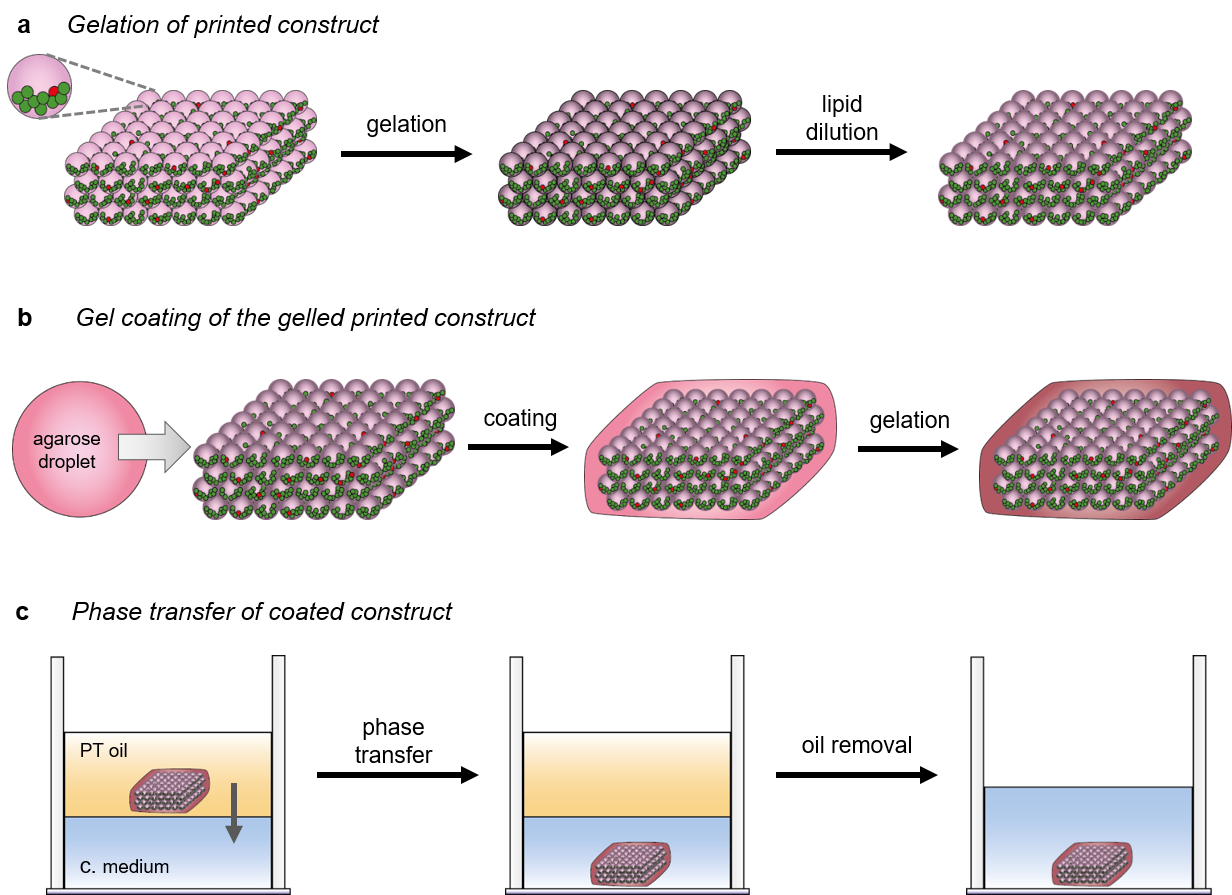


**Supplementary Figure 1:** Gel encapsulation and phase transfer of printed cellular constructs**. a,** The printed construct is first gelled within the print oil by cooling from ambient temperature and standing at 4°C for 25 min. The lipid of the print oil is then diluted to ~15 μM, by repeated silicone oil washes at room temperature, leaving the construct free of an outer lipid coating. **b**, Gel encapsulation of a printed construct. The surface of the gelled construct is wetted with a pipetted agarose droplet. The agarose enveloped structure is then gelled within the oil (4°C, 25 min). **c**, Phase transfer. The gelled encapsulated construct is warmed to ambient temperature and then moved into the upper phase of a two-phase column of oil-above-culture-medium and sinks by gravity through the interface shedding the oil phase. The oil is removed from the container, which is then topped up with culture medium and stored in a cell incubator.


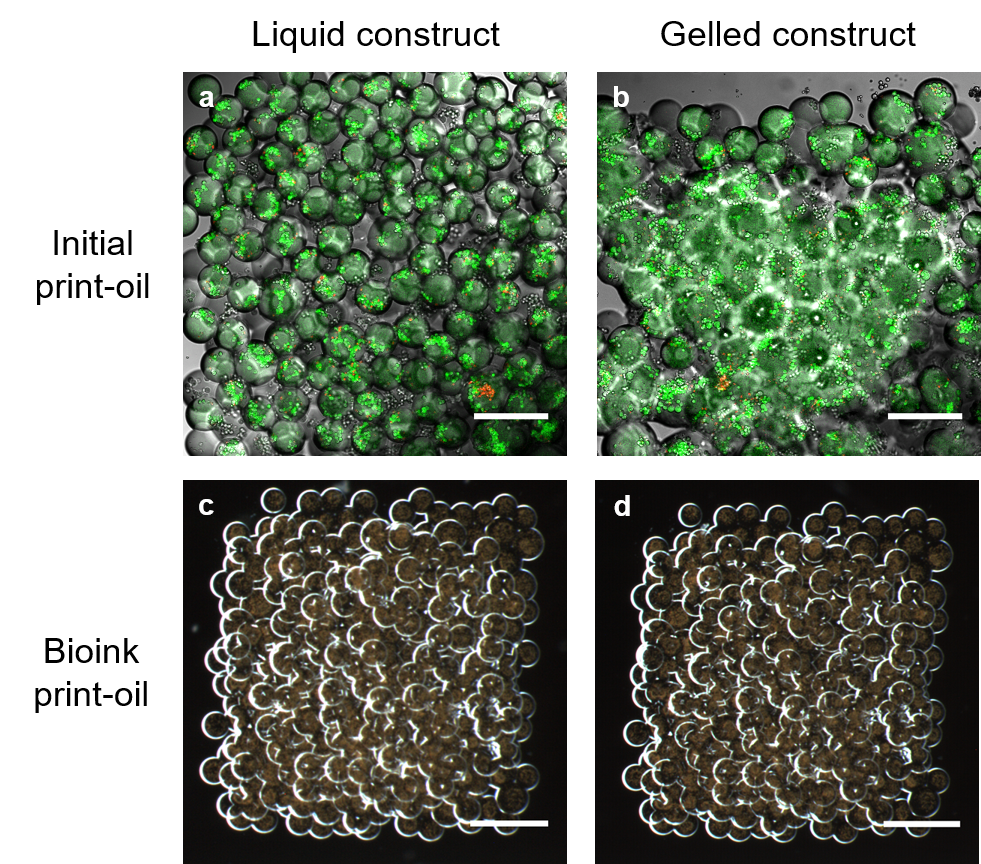


**Supplementary Figure 2:** The effect of the oil composition on construct gelation. **a-d,** Micrographs of printed droplet constructs containing either cells or polystyrene micro-beads (ø 5 μm, Sigma-Aldrich) in different physical states: **a**, **c,** fluid constructs imaged immediately after printing and; **b**, **d,** gelled constructs imaged after temperature induced gelation (4°C, 60 min). **a-b**, A live/dead stained HEK-293T cellular construct fabricated in the initial print oil mix (1.2 mM DPhPC in 1:1 v:v hexadecane: silicone oil AR20) displayed a defined printed structure (**a**), which was lost upon gelation because the oil froze during cooling (**b**). **c**-**d**, A bead-laden droplet construct printed in the standard bioink print oil (1.5 mM DPhPC in 35:65 v:v undecane: silicone oil AR20) with a defined square geometry (**c**), which upon gelation showed no discernible loss of structure (**d**). Images are: **a**-**b**, composite bright-field and fluorescence confocal micrographs and; **c**-**d**, dark-field micrograph. Scale bars = 300 μm.


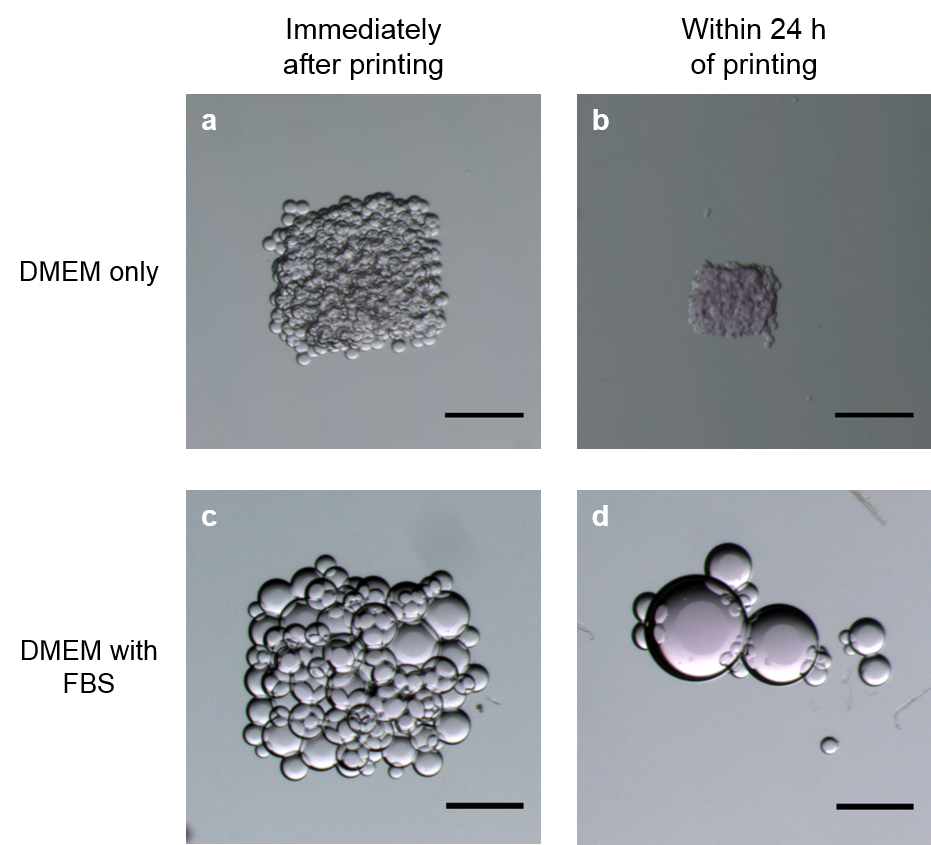


**Supplementary Figure 3:** Time-dependant coalescence of cell-free constructs printed without stabilising agents. **a**-**d**, Bright-field micrographs of printed constructs comprising only of culture medium observed: **a**, **c**, immediately after printing and: **b, d**, within 24 h of printing. **a**, A construct printed with DMEM which omitted FBS, displayed no coalescence events during printing and packed similarly to printed constructs containing isotonic salt solutions. **b**, The construct of **a** after 23 h, here the structure was retained but had dehydrated. **c**, A construct printed with DMEM containing 10% v/v FBS. During printing the droplets coalesced producing non-uniformly sized construct droplets. **d**, The construct of **c** after 14 h. The structure was reduced to less than 20 droplets by multiple coalescence events. Scale bars = 300 μm.


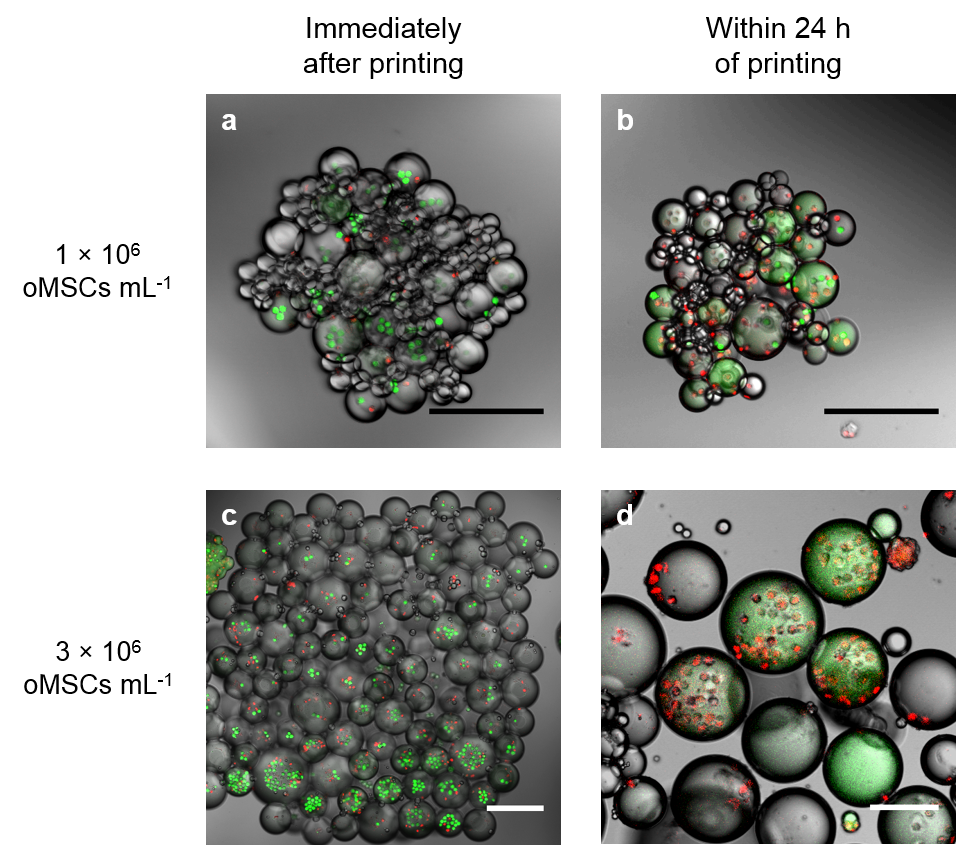


**Supplementary Figure 4:** Time-dependent coalescence of cellular constructs printed without stabilising agents**. a**-**d**, Composite bright-field and fluorescence confocal micrographs of live/dead stained oMSC constructs comprised of a bioink of serum-free culture medium. Live and dead cells were stained with calcein-AM (green) and propidium iodide (red), respectively. **a**, **c**, Constructs immediately after printing, containing either: **a**, 1 × 10^6^ or **c**, 3 × 10^6^ cells mL^‑1^. Construct droplets had coalesced during printing giving non-uniform droplet sizes. **b**, **d**, Constructs of **a** and **c** respectively after: **b**, 6 h and **d**, 22 h. Further coalescence had occurred and the majority of the cells were dead. The construct in **d** also displayed very poor droplet–droplet cohesion. Scale bars are: **a**-**c**, 250 μm and: **d** 100 μm.


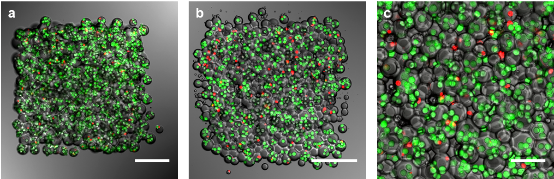


**Supplementary Figure 5:** Enhanced stability and droplet packing of cellular constructs incorporating Fmoc-dipeptides**.** **a**-**c**, Composite bright-field and fluorescent confocal micrographs of live/dead stained HEK-293T cellular constructs immediately after printing. The bioink was comprised 1 mM Fmoc-XX in 70% v/v Opti‑MEM® with 2 × 10^6^ cells mL^‑1^. **a**-**b**, Constructs were printed as 11 × 14 droplet sheet for 2 to 3 layers and showed no coalescence (compare Supplementary Figure 4). **c**, Magnified image showing droplet-droplet packing within construct of **b**, showed higher droplet cohesion than in constructs without Fmoc-XX (compare Supplementary Figure 4). Two cells were present on average per droplet. Scale bars are: **a**-**b**, 200 μm and; **c**, 75 μm.


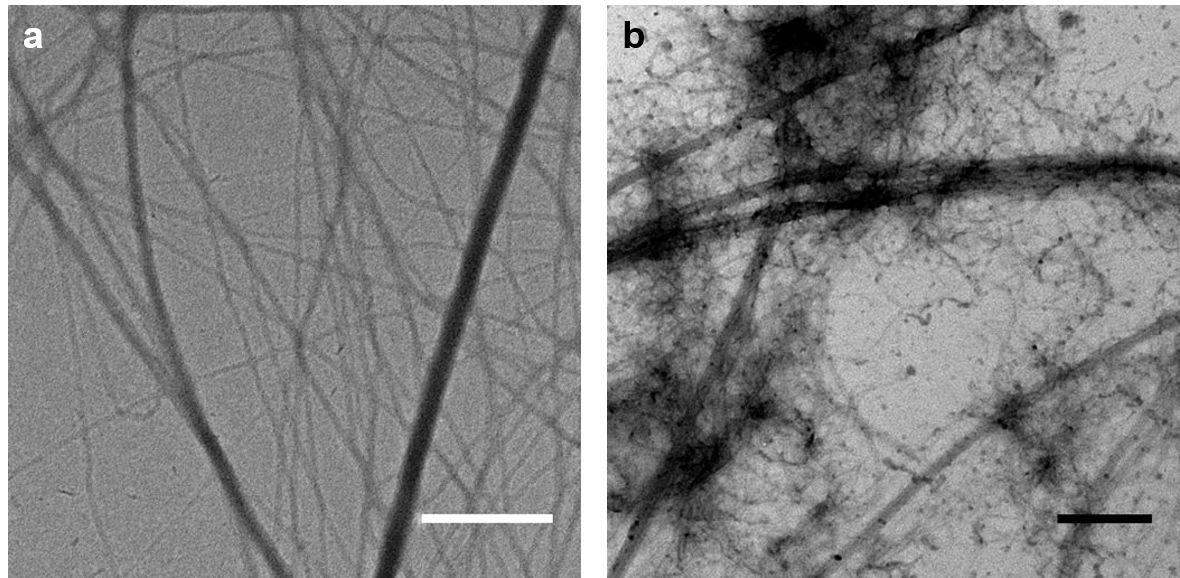


**Supplementary Figure 6:** Transmission electron micrographs of gelled bioink. **a**, A 1:1 v:v mixture of 10 mM Fmoc-IG to 10 mM Fmoc-FF (referred to as 10 mM Fmoc-XX) gelled by dropwise addition of hydrochloric acid. Visible is a mixture of thin peptide protofibrils and mature fibrils sized *d* = ~30 to 40 nm and *d* = ~100 nm respectively^12^. **b**, A 1:1 v:v mixture of 10 mM Fmoc-XX to 15 mg mL^-1^ ultra-low gelling temperature agarose gelled by cooling (4°C, 10 min) and subsequently stained with 2% v/v uranyl acetate (pH ~4.3, 1 min) and then washed with Milli‑Q® water. Large fibre bundles (*d* = ~80 nm) and smaller protofibrils (*d* = ~ 20 to 30 nm) were observed. Both samples were imaged with a FEI Tecnai™ T12 transmission electron microscope. Scale bars = 250 nm.


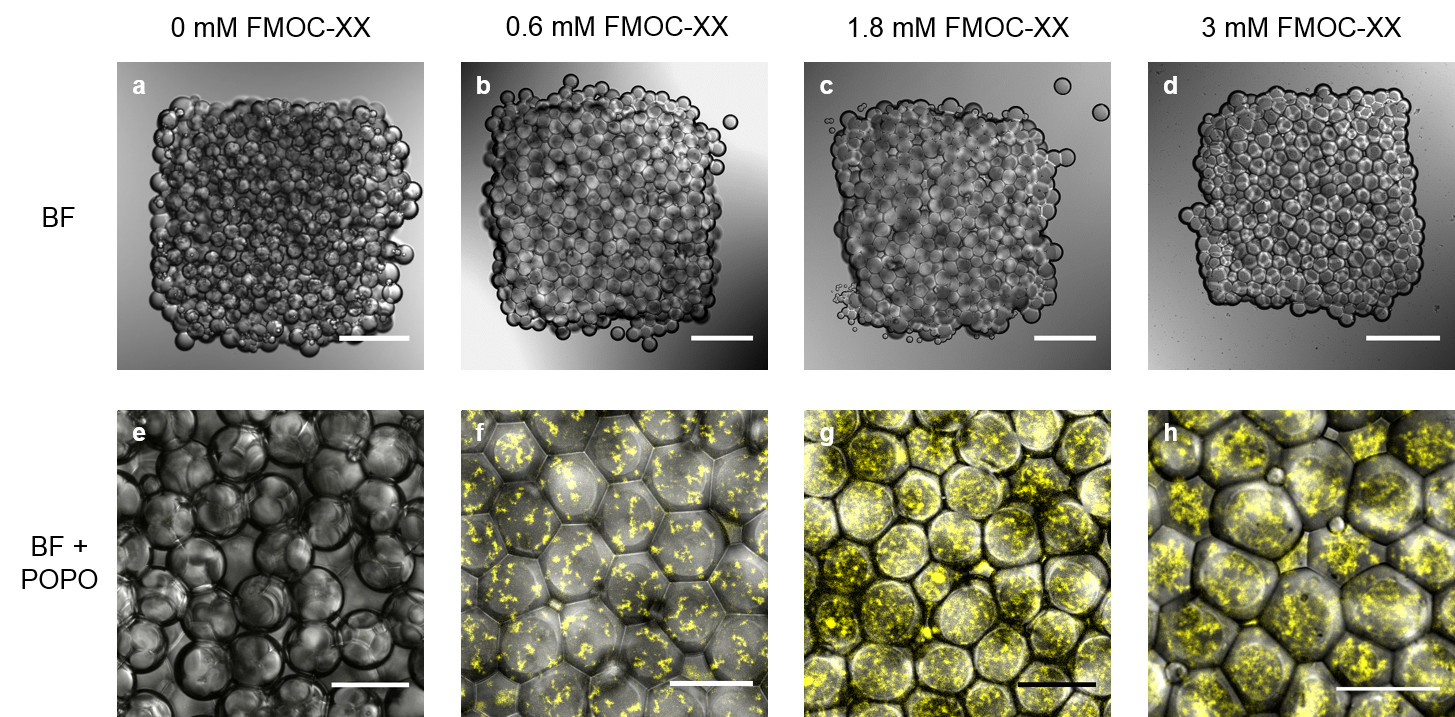


**Supplementary Figure 7:** The effect of Fmoc-dipeptides on 3D droplet packing. **a**-**h**, Confocal micrographs of constructs comprising 70% v/v Opti-MEM® with 0 to 3 mM Fmoc-XX and 5 μm POPO-3 iodide, imaged immediately after printing. **a**-**d**, Bright-field micrographs of printed constructs containing Fmoc-XX, used as a stabilising agent, at increasing concentrations: **a** 0 mM; **b** 0.6 mM; **c** 1.8 mM and; **d** 3 mM. **e**-**f**, Composite bright-field and fluorescence micrographs of the constructs of **a**-**d** respectively, magnified to display the extent of Fmoc-dipeptide aggregation (yellow, POPO-3 iodide stain) and the packing of the droplets. The inclusion of Fmoc-dipeptides resulted in reduced oil spaces between construct droplets and decreased contact angles between droplets. Scale bars are: **a**-**d**, 250 μm and **e**-**h**, 75 μm.


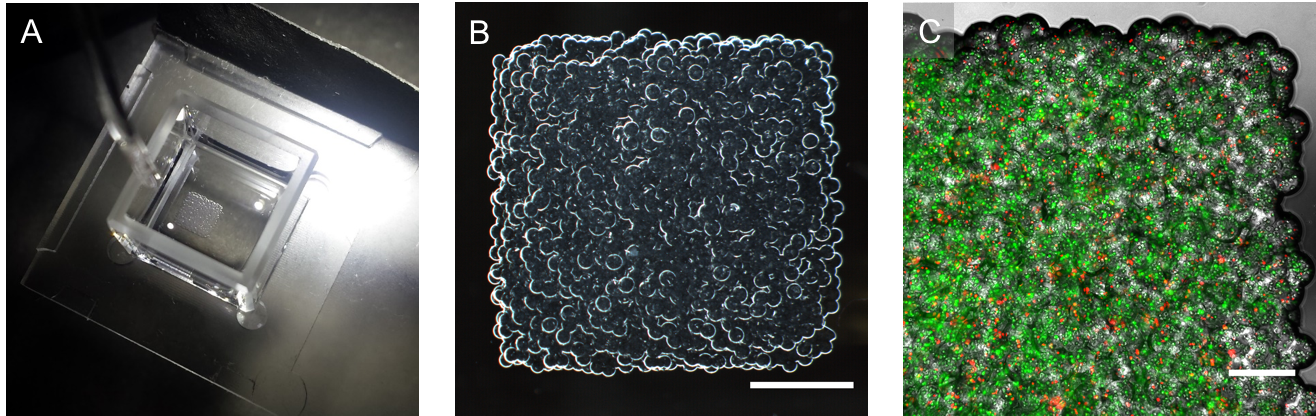


**Supplementary Figure 8**: A 3 mm wide printed cellular cuboid. **a**, Photograph of a glass cuvette containing a printed construct with 3 × 3 × 0.5 mm dimensions in oil. **b**, Dark-field micrograph of the printed construct of (**a**) in oil. **c**, Fluorescence micrograph of the printed cellular construct of (**a**) in oil, with live/dead stained cells (live, calcein-AM, green; and dead, propidium iodide, red). Scale bars are: **b**, 1 mm and **c**, 300 μm.


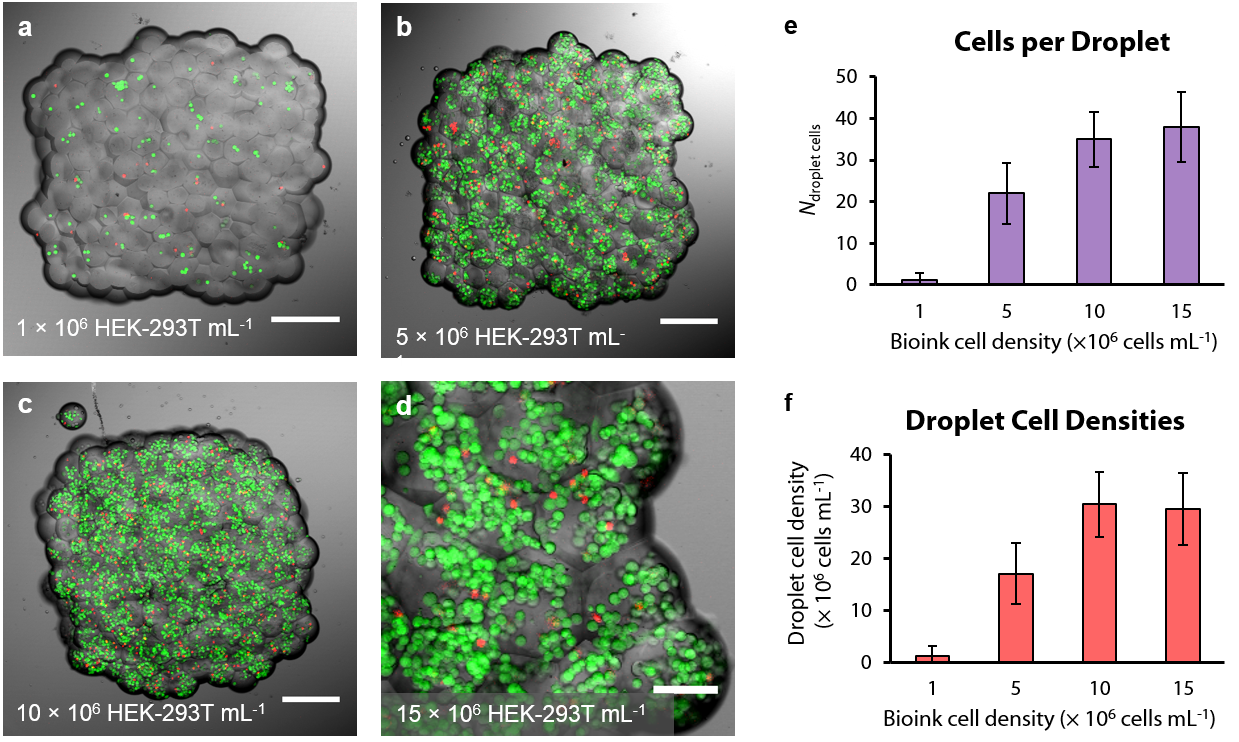


**Supplementary Figure 9:** The effect of bioink cell density on printed droplet cell density. **a**‑**d**, Composite bright-field and fluorescence confocal micrographs of HEK-293T cellular constructs, printed at different bioink cells densities: **a**, 1 × 10^6^; **b**, 5 × 10^6^; **c**, 10 × 10^6^ and; **d**, 15 × 10^6^ cells mL^-1^. The constructs were created with a bioink containing live/dead dyes and were imaged immediately after printing while still in bulk oil. Images show: **a**-**c**, the entire construct or **d**, cells compartmentalised within droplets. **e**, The average cell number per droplet for constructs **a**-**d**, calculated from manual counts, here the average droplet sizes were: **a**, 120; **b**, 135; **c**, 130 and; **d**, 135 μm diameter. **f,** The average cell densities of droplets within constructs **a**-**d,** calculated as the average cell occupancy of droplets divided by the average droplet volume. Error bars represent the standard deviations of the cell count (**e**) or the compound errors of the counts and droplet volumes (**f**). Scale bars are: **a**-**c**, 250 μm and; **d** 75 μm.


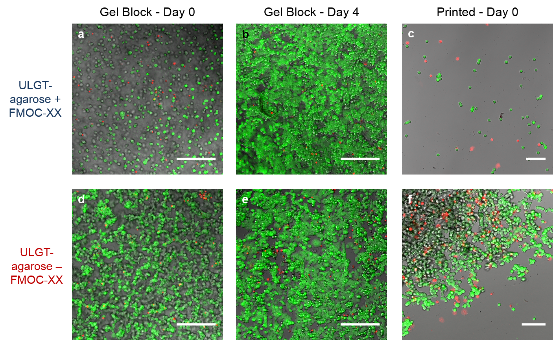


**Supplementary Figure 10:** HEK-293T cell growth within gelled bioink and viability after printing using two bioink formulations. **a**-**b, d-e,** Composite bright-field and fluorescence confocal micrographs of live/dead stained HEK-293T cells in blocks (5 μL) of gelled bioink imaged: **a**, **d**, immediately after gelation (4˚C, 30 min) and; **b**, **e** after 4 d in culture. For both bioink solutions, over 4 d the cells went from single cells or small aggregates to confluent cell masses indicative of proliferation. **c**, **f**, Composite bright-field and fluorescence confocal micrographs of live/dead stained HEK-293T immediately after being ejected as droplets in air (“printed”) into culture medium. Viabilities were determined from manual cell count values to be: **c**, 83% and; **f**, 84%, indicating the piezo-actuated cell ejection was not immediately detrimental to HEK-293T cells. The bioinks contained 5 × 10^6^ cells mL^-1^ and ultra-low gelling temperature (ULGT) agarose (12 mg mL^-1^): **a**-**c**, with 1.0 mM Fmoc-XX or **d**-**f**, without Fmoc-XX. Scale bars are **a**-**b**, **d**-**e**, 200 μm; and **c**, **f**, 100 μm.


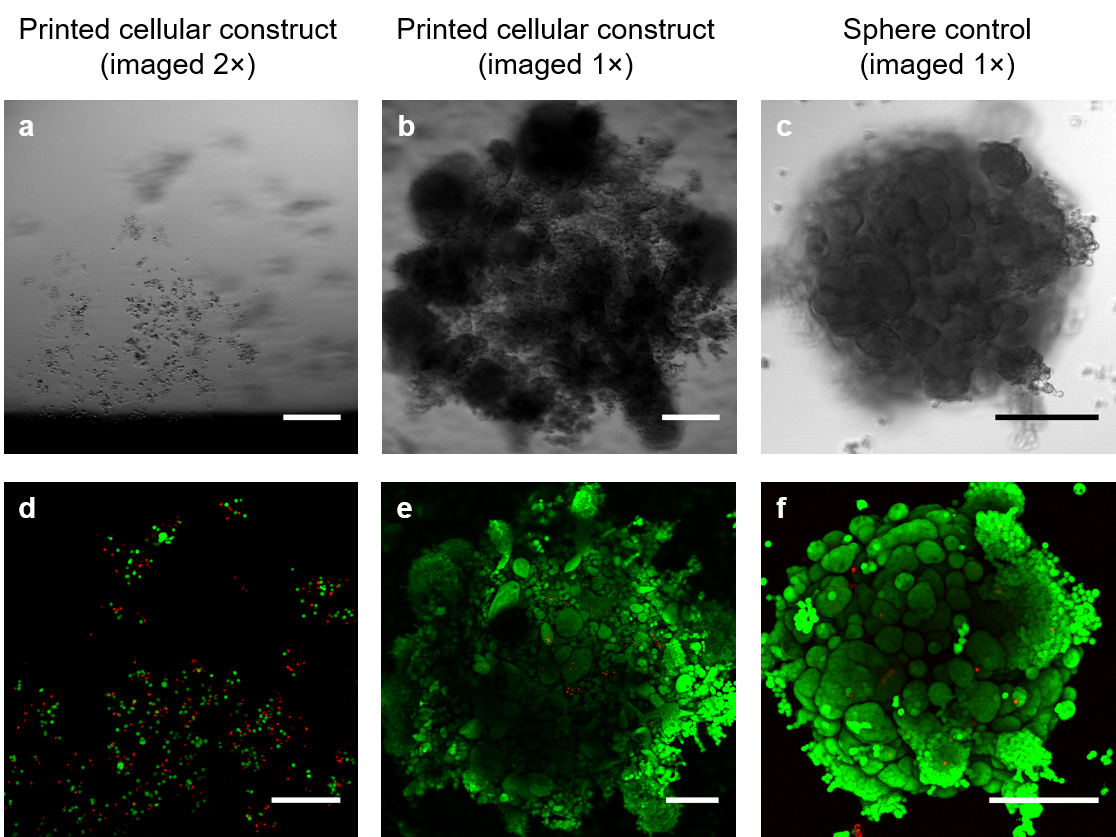


**Supplementary Figure 11:** Effect of radiant exposure from confocal microscopy on HEK-293T cell development**. a**-**f** Images of HEK-293T cellular constructs live/dead stained after 7 days of culture. Here, each construct was made from a bioink containing 15 × 10^6^ cells mL^-1^ and 15 μg mL^-1^ type I collagen. **a**, **d,** A printed cellular construct which was previously imaged on day 0, showed arrested HEK-293T proliferation on day 7, with discrete single cells visible. **b**, **e,** A printed cellular construct previously unimaged displayed dense masses of viable HEK-293T cells. **c**, **g,** A non-printed cellular construct after 7 d in culture. The control sample was made as a droplet (0.2 μL) of bioink solution in oil that was gelled (4˚C, 20 min) and transferred to culture medium. The construct was previously unimaged and showed cell proliferation and high cell viabilities. Images were: **a**-**c** bright-field confocal micrographs and; **d**-**f**, images of 3D reconstructions of live/dead-stained cells. Scale bars = 250 μm.


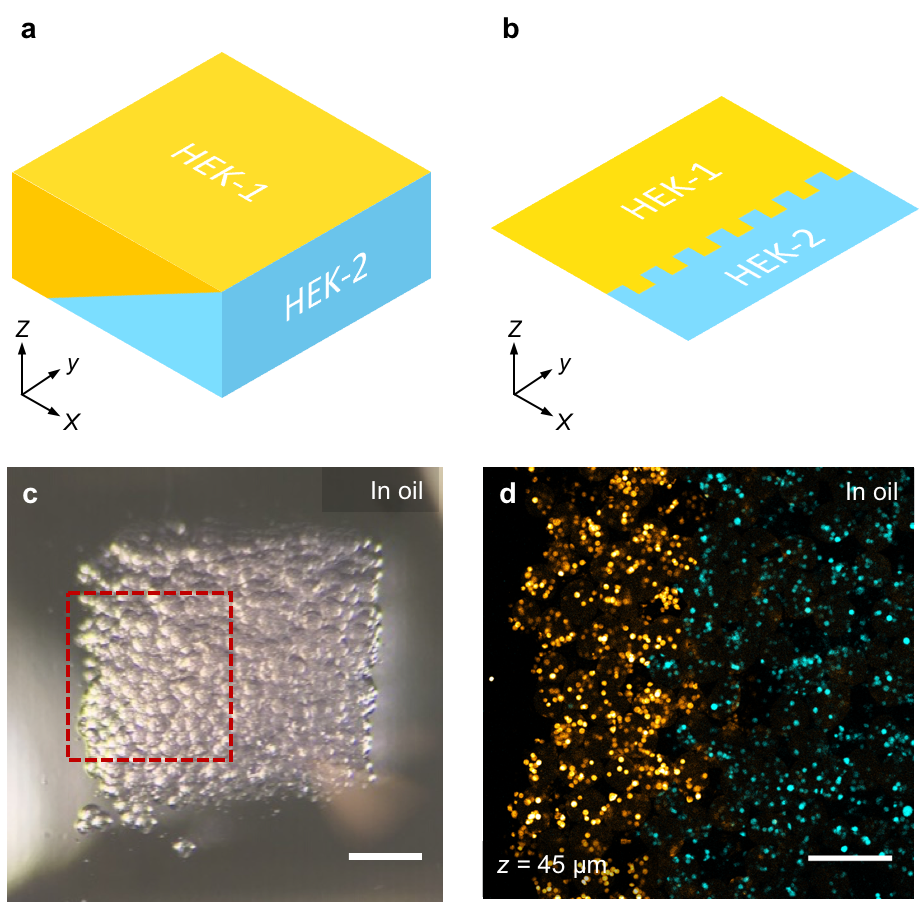


**Supplementary Figure 12**: 3D model and images of a 3D printed cellular junction with a diagonal interface in the *x*-*z* plane. **a**, A 3D model of the junction between two cell populations (HEK 1, yellow-orange and HEK 2, cyan), with the cellular interface in the vertical (*x*-*z*) plane. **b**, A representative cross section of the 3D model with an interdigitated interface between the cell populations; each “finger” is one droplet resolution. **c**, Bright-field micrograph of the printed junction (21 × 24 × 7 droplets) in oil. The red square overlay represents the region that was imaged by fluorescence confocal microscopy as shown in d. **d**, A fluorescence micrograph of the base of the printed junction showing distinct cell populations in the horizontal (*x*-*y*) plane with one droplet overlap at the interface. Scale bars are: **c**, 500 µm and **d**, 250 µm.


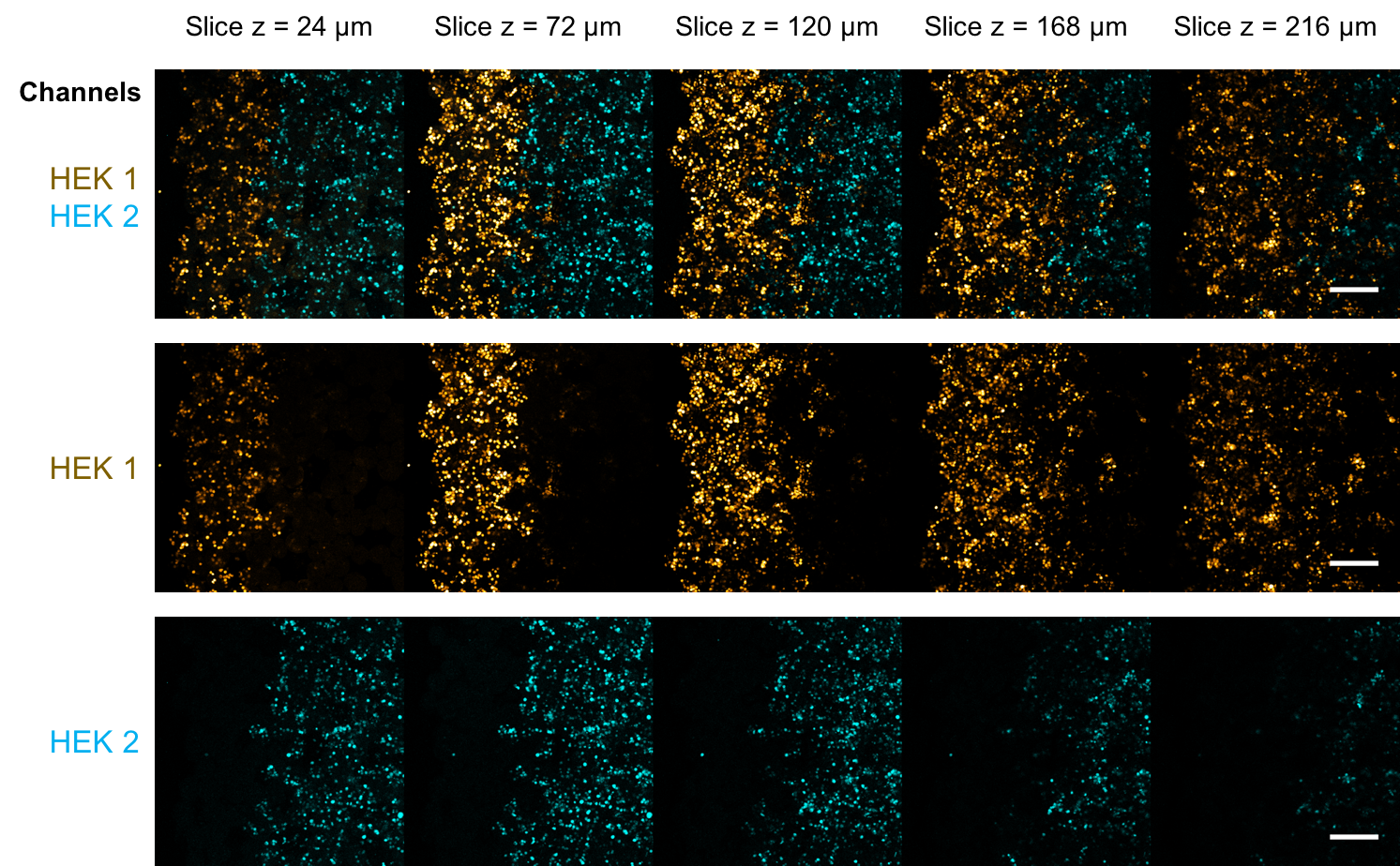


**Supplementary Figure 13**: Visualisation of the 3D cellular pattern of a 3D printed junction with a diagonal interface in the vertical (*x*-*z*) plane (see Supplementary Figure 11). Each of the three rows is an image montage of five fluorescence confocal micrographs, depicting the cellular junction (21 × 24 × 7 droplets) in oil at increasing vertical (*z*) heights (increments of 48 µm). The top row shows both fluorescent cell populations (HEK 1, orange-yellow; and HEK 2, cyan), while the lower two rows show a single cell population only. As the *z*-height is increased, it can be visualised within the horizontal (*x*-*y*) plane of the construct that the HEK 1 population increases and spreads right along the *x*-axis, while the HEK 2 population decreases and retreats right along the *x*-axis. This demonstrates the interface between the cell populations is at a diagonal in the *x*-*z* plane. Scale bar = 250 µm.


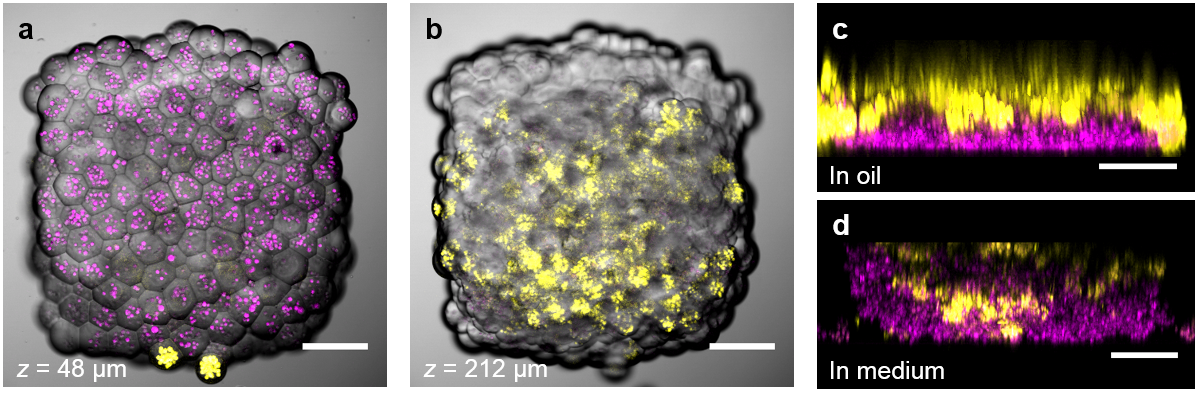


**Supplementary Figure 14:** A layered osteoblast and chondrocyte printed construct. **a-b**, Composite bright-field and fluorescence confocal micrographs of a layered cellular construct showing different cell layers: **a**, lower chondrocyte layer (4 droplets thick) at 48 μm and; **b**, upper osteoblast layer (4 droplets thick) at 212 μm. Chondrocytes (purple) and osteoblasts (yellow) were stained with Deep Red and Red CMPTX CellTracker™ dyes, respectively. **c**-**d**, Side-on image of 3D reconstructions of a layered cellular constructs, **c**, immediately after printing and **d**, after transfer to culture medium. **c**, Defined chondrocyte and osteoblast layers are visible after printing. **d**, After phase transfer, the construct had flexed but the osteoblast layer remained present above the chondrocyte layer after phase transfer. Scale bars = 250 μm.


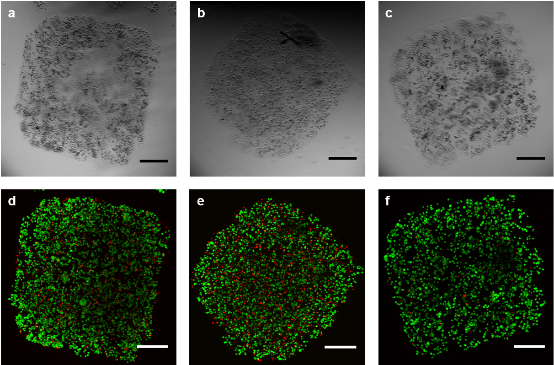


**Supplementary Figure 15:** Printed cellular constructs after transfer to culture medium. **a**-**c**, Bottom-up bright-field confocal micrographs of printed cellular constructs, with square geometries, in culture medium. The constructs were printed with a bioink containing 15 μg mL^-1^ type I collagen and either: **a**-**b**, 15 × 10^6^ HEK-293T cells mL^-1^ or; **c**, 15 × 10^6^ oMSCs mL^-1^. **d**-**f**, Images of 3D reconstructions of live/dead stained cells within phase transferred constructs **a**-**c**, respectively. The constructs retained their square shape after phase transfer. Scale bars = 250 μm.


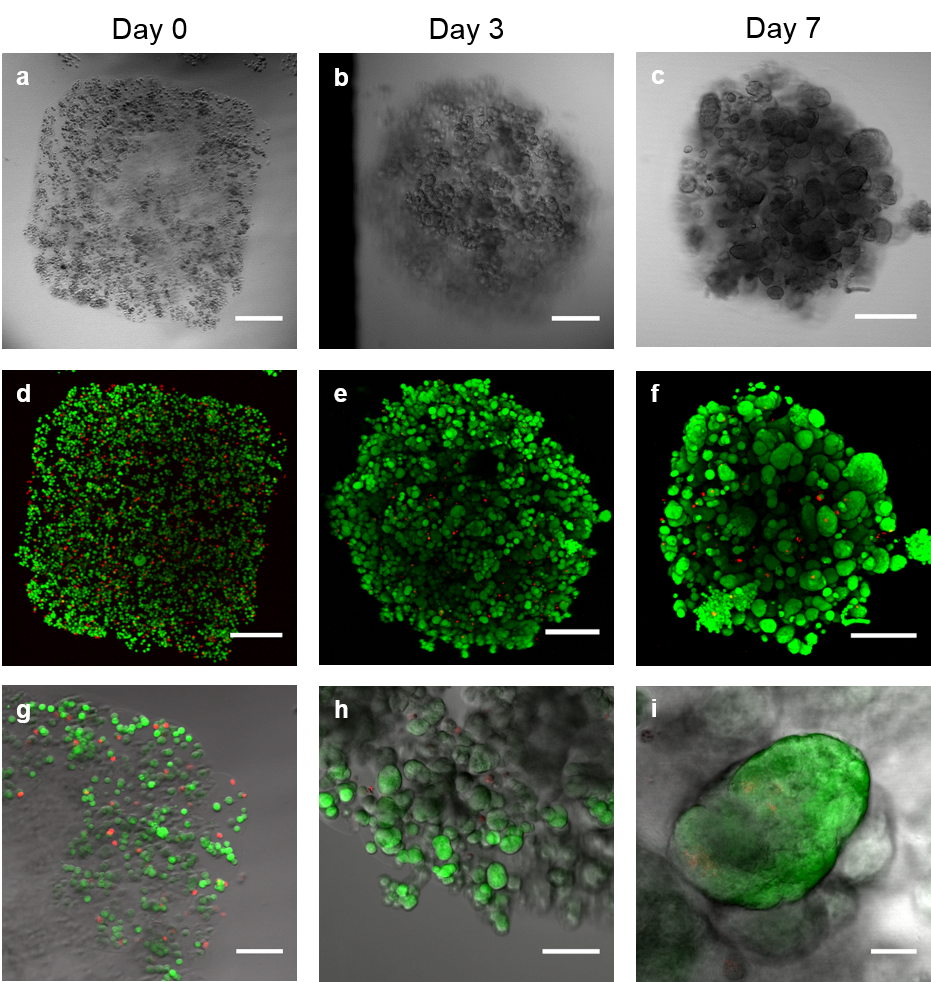


**Supplementary Figure 16:** Development of printed HEK-293T cells within constructs over 7 d. **a**-**i**, Images of individual printed HEK-293T cellular constructs over 7 d of cell culture. Constructs were printed with a bioink containing 15 × 10^6^ cells mL^-1^ and 15 μg mL^-1^ type I collagen. Constructs were imaged on day 0 (left-hand column, *n* = 4 constructs), day 3 (middle column, *n* = 4 constructs) and day 7 (right-hand column, *n* = 4 constructs). **a-c**, Bright-field confocal micrographs of constructs, here the constructs became dark and opaque over 7 d as the cells proliferated. **d**-**f**, Images of 3D reconstructions of live/dead stained cellular constructs, which indicate a high viability throughout the course of culture. **g**-**i**, Composite bright-field and fluorescence micrographs showing magnified sections of printed cellular constructs. The cells had proliferated from individual entities to dense cell clusters. **i**, Cell clusters on day 7 were up to 140 μm wide. Scale bars are: **a**-**f**, 250 μm; **g**-**h**, 100 μm and; **i**, 30 μm.


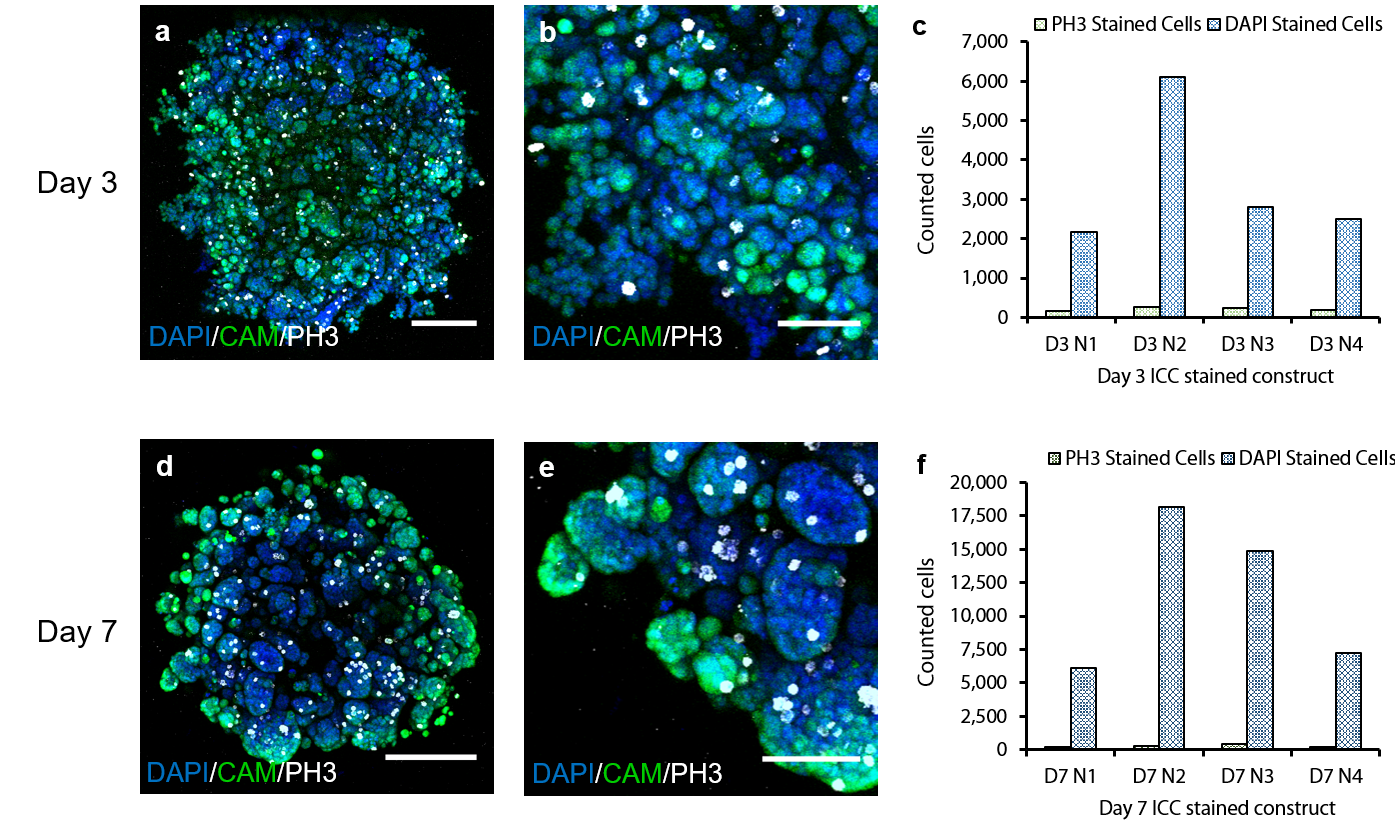


**Supplementary Figure 17:** Printed HEK-293T cells immunostained after culture. **Left hand and middle column,** Images of 3D reconstructions of independent HEK-293T cellular constructs which were immunostained after culture. These constructs were printed at 15 × 10^6^ cells mL^-1^ in a bioink supplemented with 15 μg mL^-1^ type I collagen. Cells were stained for cell nuclei (DAPI, **blue**); phospho-histone 3 (ICC, PH3, **white**) and viable cells (calcein‑AM, CAM, **green**). **a**, **d,** Images of an entire construct on: **a,** day 3 and; **d,** day 7. **b**, **e,** Magnified sections of constructs showing internal cell aggregates on: **b,** day 3 (construct **a**) and; **e,** day 7 (construct **d**). **Right hand column,** Counted cells within ICC-stained constructs on: **c,** day 3 (D3) and; **f,** day 7 (D7). Constructs were labelled N1 to N4 for both the day 3 and 7 sets. Plotted here are the resolved counts of DAPI-stained cells (**blue**) and the unmodified counts of PH3-stained cells (**green**). Scale bars are: **a**, **d**, 250 μm and; **b**, **e**, 100 μm.


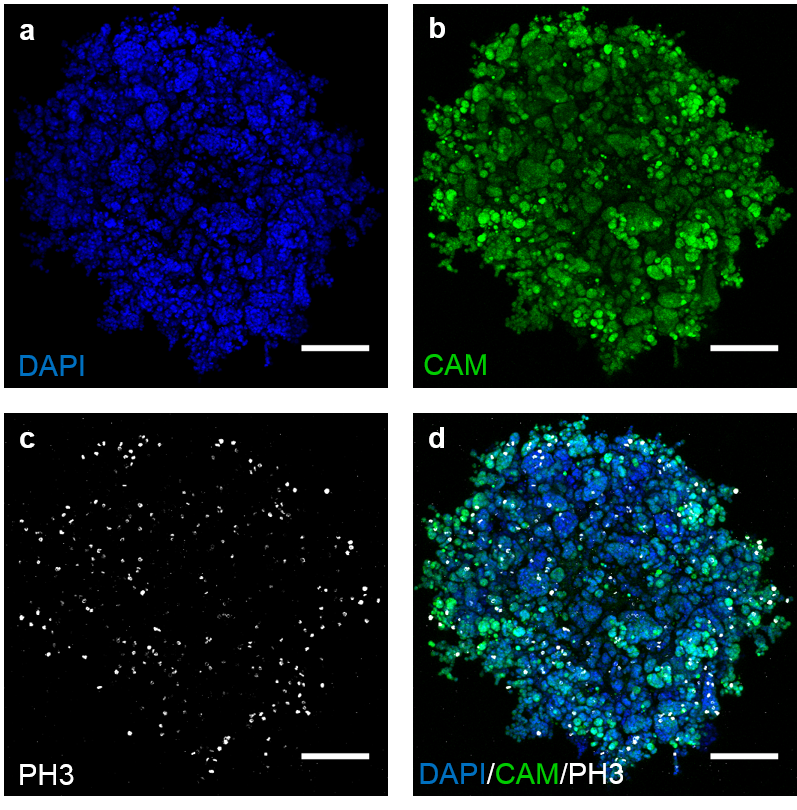


**Supplementary Figure 18:** Printed HEK-293T cells after 3 d in culture. **a-d,** Images of the different channels of a 3D reconstruction of a multi-stained HEK-293T cellular construct, printed with a bioink containing 15 × 10^6^ cells mL^-1^ and 15 μg mL^-1^ type I collagen. The channels represent the fluorescent emissions resulting from the different staining methods: **a**, nuclear staining (DAPI, **blue**); **b**, live cell staining (calcein‑AM, CAM, **green**); **c**, immunostaining (anti-phospho-histone 3 antibodies, PH3, **white**). **d**, Composite image of all three channels**.** Scale bars = 250 μm.


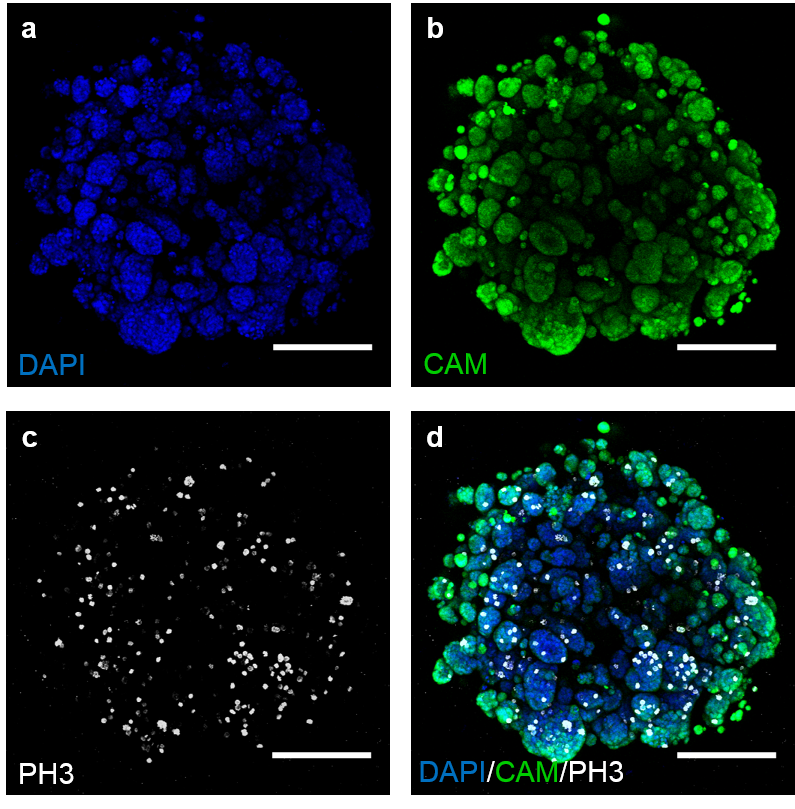


**Supplementary Figure 19:** Printed HEK-293T cells after 7 d in culture. **a-d,** Images of the different channels of a 3D reconstruction of a multi-stained HEK-293T cellular construct, printed with a bioink containing 15 × 10^6^ cells mL^-1^ and 15 μg mL^-1^ type I collagen. The channels represent the fluorescent emissions resulting from the different staining methods: **a**, nuclear staining (DAPI, **blue**); **b**, live cell staining (calcein‑AM, CAM, **green**); **c**, immunostaining (anti-phospho-histone 3 antibodies, PH3, **white**). **d**, Composite image of all three channels**.** Scale bars = 250 μm.


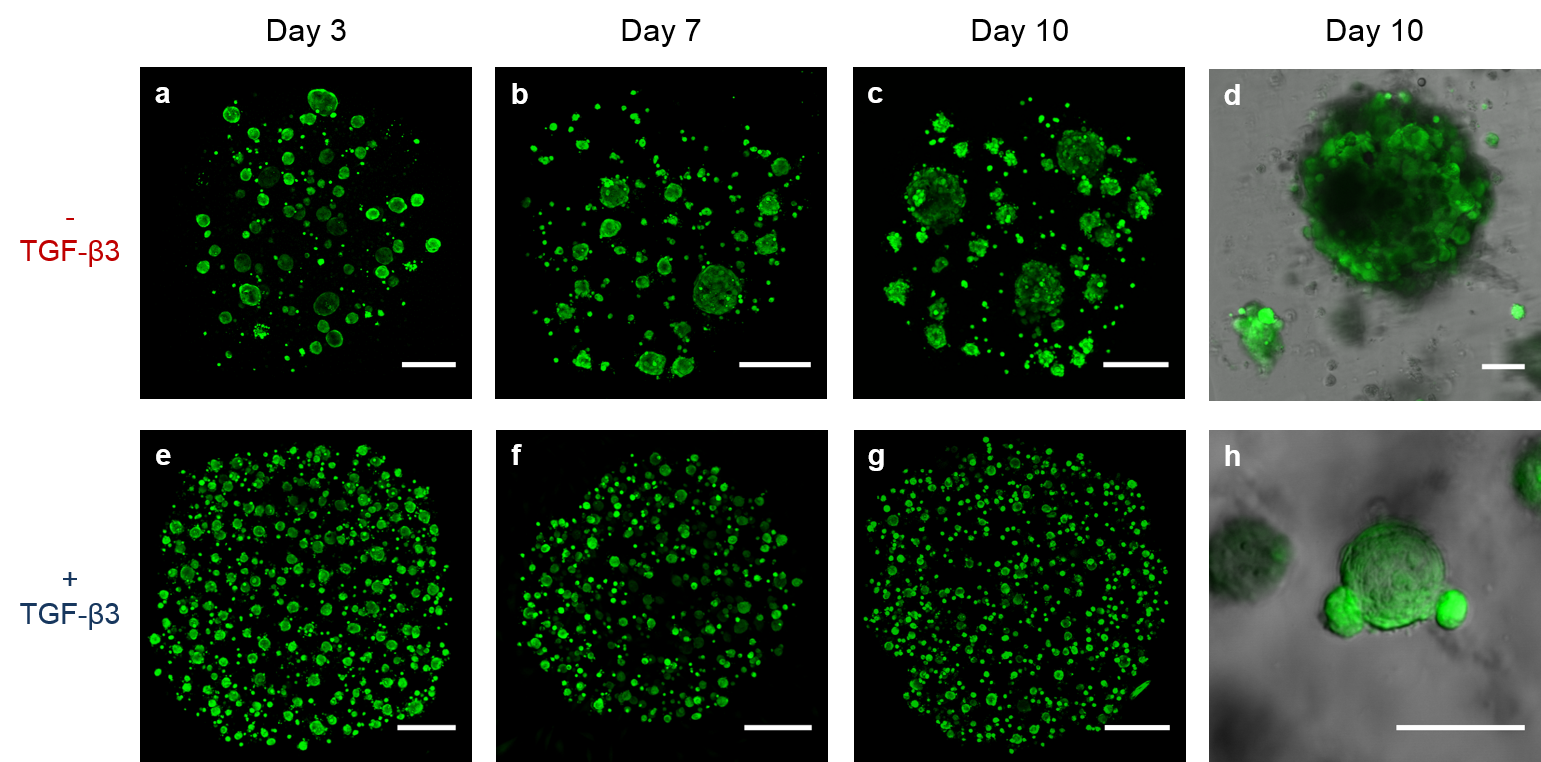


**Supplementary Figure 20:** Development of printed oMSCs within constructs cultured with and without TGF-β3. **a-h,** Images of printed oMSC over 10 d culture without TGF-β3 (top row) or with TGF-β3 (bottom row). Constructs were printed from a bioink with a cell density of 15 × 10^6^ cells mL^-1^ and supplemented with 15 μg mL^-1^ type I collagen. **a**-**c**, **e**-**g,** Images of 3D reconstructions of live/dead stained cellular constructs on: **a**, **e**, day 3; **b**, **f**, day 7 and; **c**, **g**, day 10. **d**, **h**, Composite bright-field and fluorescence confocal micrographs of a magnified section of the oMSC-laden constructs on day 10. In the presence of TGF-β3, spheroidal cell aggregates of 25 to 60 μm diameter were regularly distributed throughout the printed construct. Without TGF-β3, fewer, larger aggregates of up to 350 μm were present. Scale bars are: **a**-**c**, **e**-**g**, 250 μm and; **d**, **h**, 50 μm.


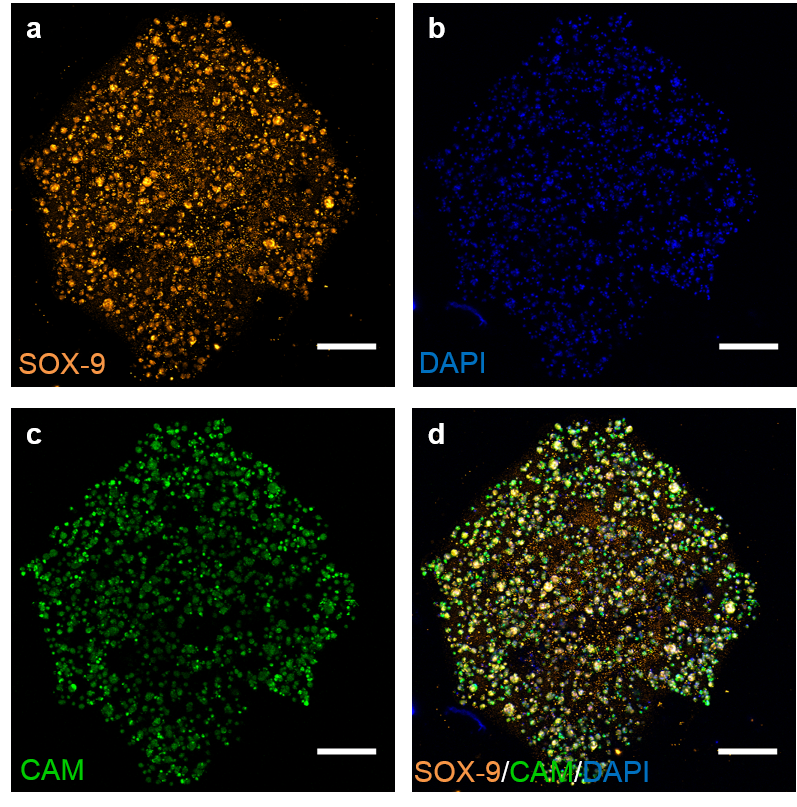


**Supplementary Figure 21:** Printed oMSCs after 3 d in culture with TGF-β3. **a-d,** Images of the different channels of a 3D reconstruction of a multi-stained oMSC containing construct, printed with a bioink containing 15 × 10^6^ cells mL^-1^ and 15 μg mL^-1^ type I collagen. The channels represent the fluorescent emission resulting from the different staining methods: **a**, immunostaining (SOX-9, **orange**); **b**, nuclear staining (DAPI, **blue**) and; **c** live-cell staining (calcein‑AM, CAM, **green**). **d**, Composite image of all three channels**.** Scale bars = 200 μm.


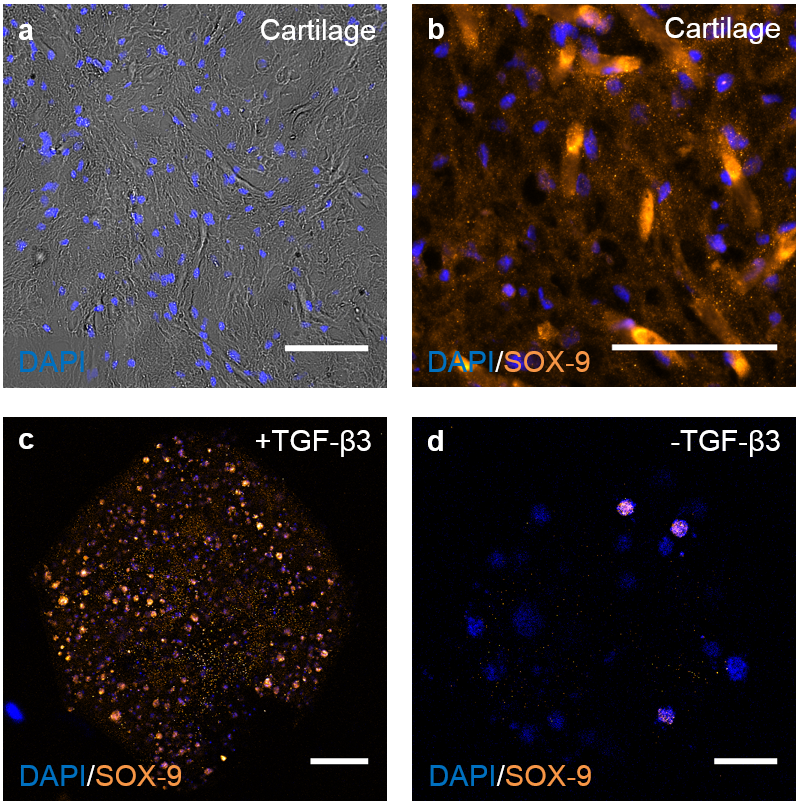


**Supplementary Figure 22:** Immunocytochemistry (ICC) performed on sectioned cartilage and printed oMSCs. **a, b** Sectioned cartilage, engineered for 35 d using oMSCs seeded within a polyglycolic acid scaffold, as a positive control for SOX-9 ICC. **a**, Composite confocal fluorescence micrograph showing both the bright-field image of the cartilage and nuclear staining (DAPI, **blue**). **b**, Confocal fluorescence micrograph showing both nuclear staining (DAPI, **blue**) and SOX-9 staining (ICC, **orange**) present in the chondrocytes within the tissue section. **c**, **d**, Confocal fluorescence micrographs of printed oMSCs after 3 d in culture: **c**, with TGF-β3 or **d**, without TGF-β3. Printed oMSCs were stained for nuclei (DAPI, **blue**) and SOX-9 (ICC, **orange**). **c**, oMSCs in printed constructs cultured with TGF-β3 show widespread staining for SOX-9. **d**, In comparison, oMSCs in printed constructs cultured without TGF-β3 show low SOX-9 signal. Scale bars are **a**, **b**, 100 μm and: **c**, **d**, 200 μm.


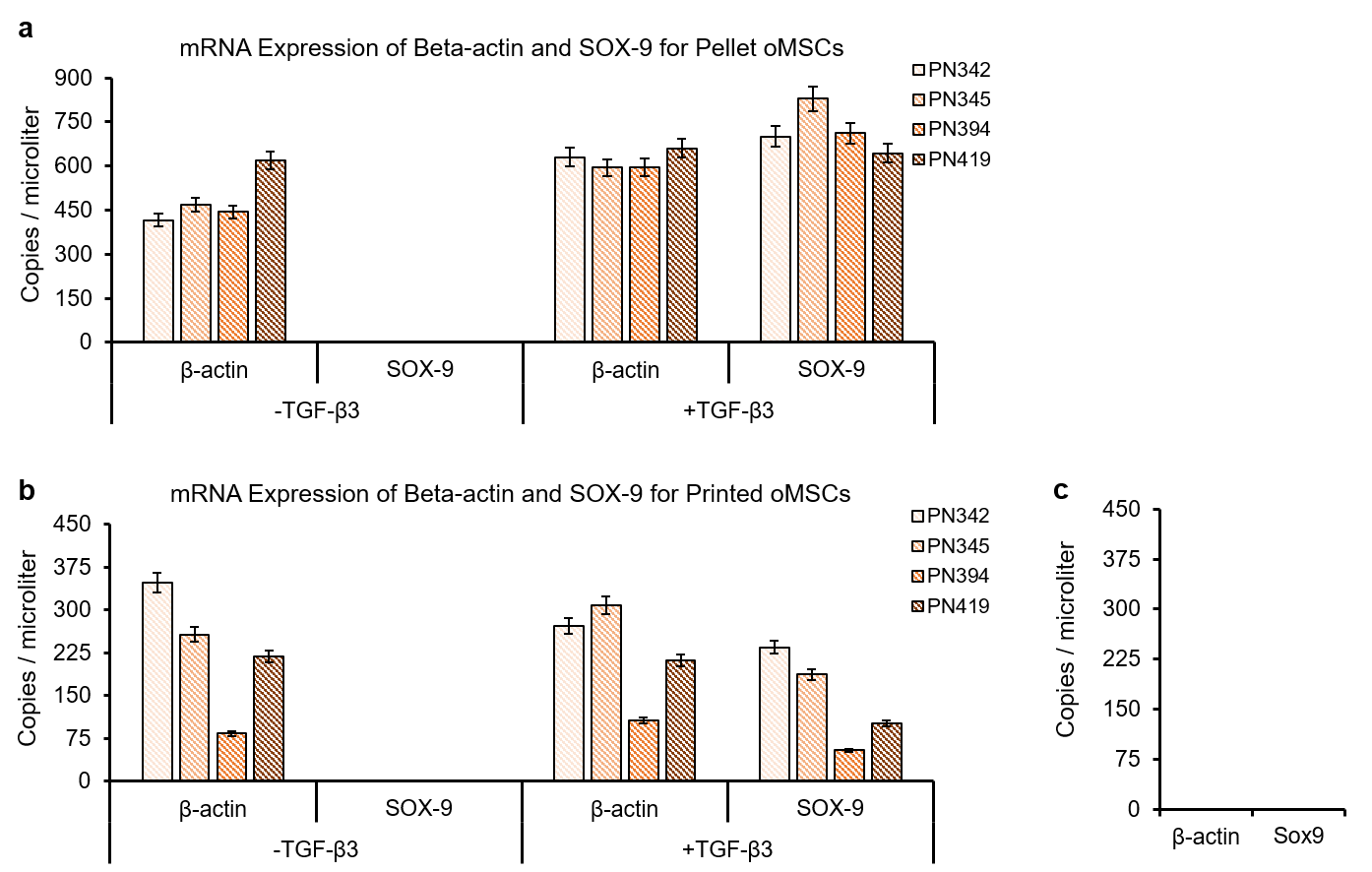


**Supplementary Figure 23:** Absolute quantification of β-actin and SOX-9 mRNA expression from printed oMSCs cultured with and without TGF-β3. **a**-**b**, mRNA expression of β-actin and SOX-9 genes measured by dPCR for oMSCs from: **a**, pellet cultures or **b**, printed constructs after 7 d of culture. Expression was measured for four different sheep; referred to as patient number (PN): 342, 345, 394, and 419. SOX-9 was only expressed at detectable levels for oMSCs cultured with TGF-β3. Error bars represent expression value ± 5%. **c**, A no template control showed no signal expression of β-actin and SOX-9.


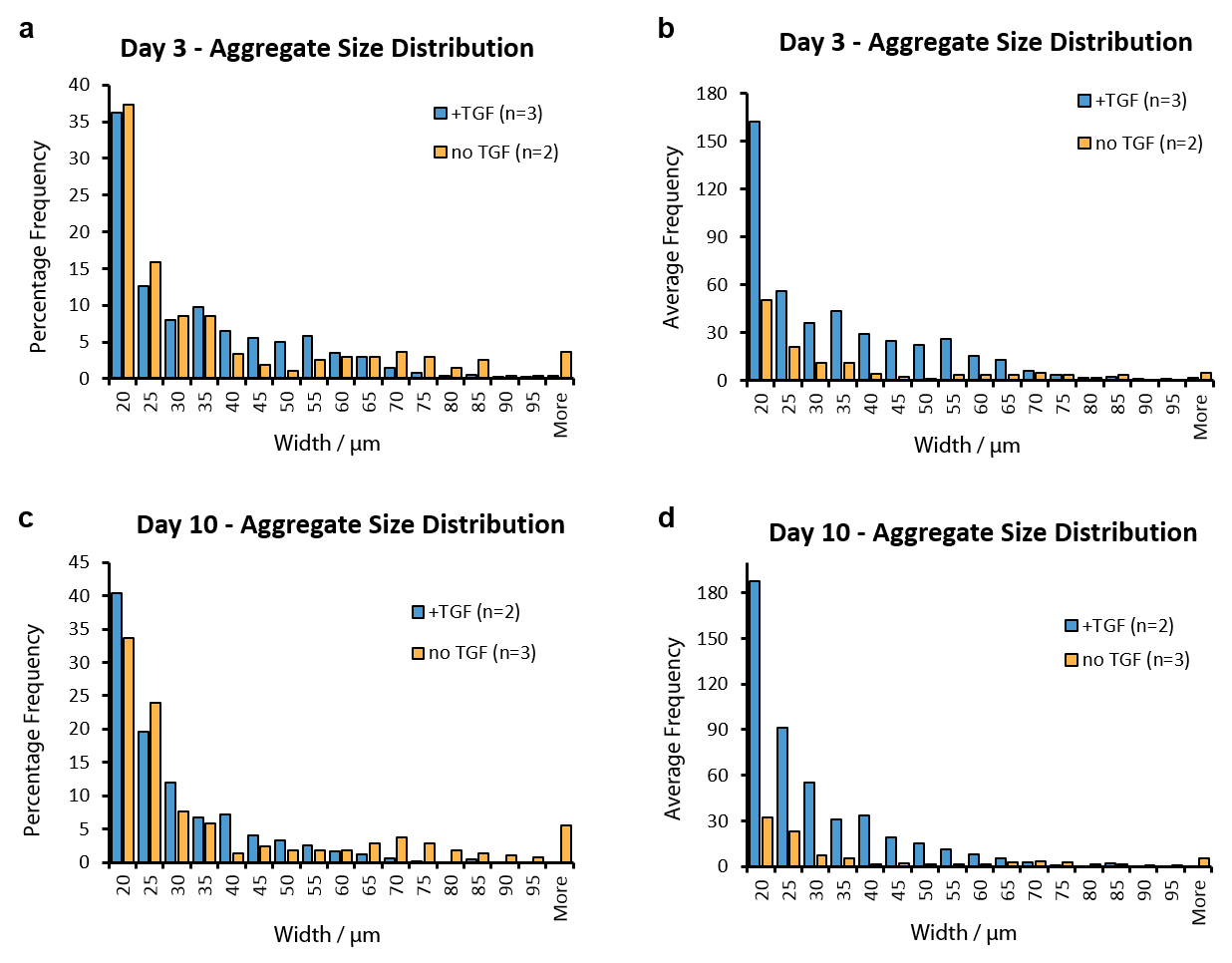


**Supplementary Figure 24:** Size distributions of printed oMSC aggregates. **a**-**d,** Histograms of cell aggregate sizes within printed oMSC-laden constructs cultured with (blue) or without (orange) TGF-β3 after **a**-**b**, 3 days and; **c**-**d**, 10 days. Shown for constructs are **a**, **c**, the proportional frequencies of aggregates and; **b**, **d**, the average frequencies of aggregates. Horizontal widths of aggregate were measured from confocal z-stack image series (Supplementary Figure 20) using the automated object counter plug-in of Fiji. Objects of size 20 μm or wider were assumed to be aggregates. The data shows that in untreated constructs there were fewer aggregates present, but a significant proportion of them were wider than 100 μm (see “More” column). Whereas for treated constructs the size distribution of aggregates was, on day 3, 93% sized 20 to 60 μm wide and on day 10, 94% sized 20 to 50 μm wide.


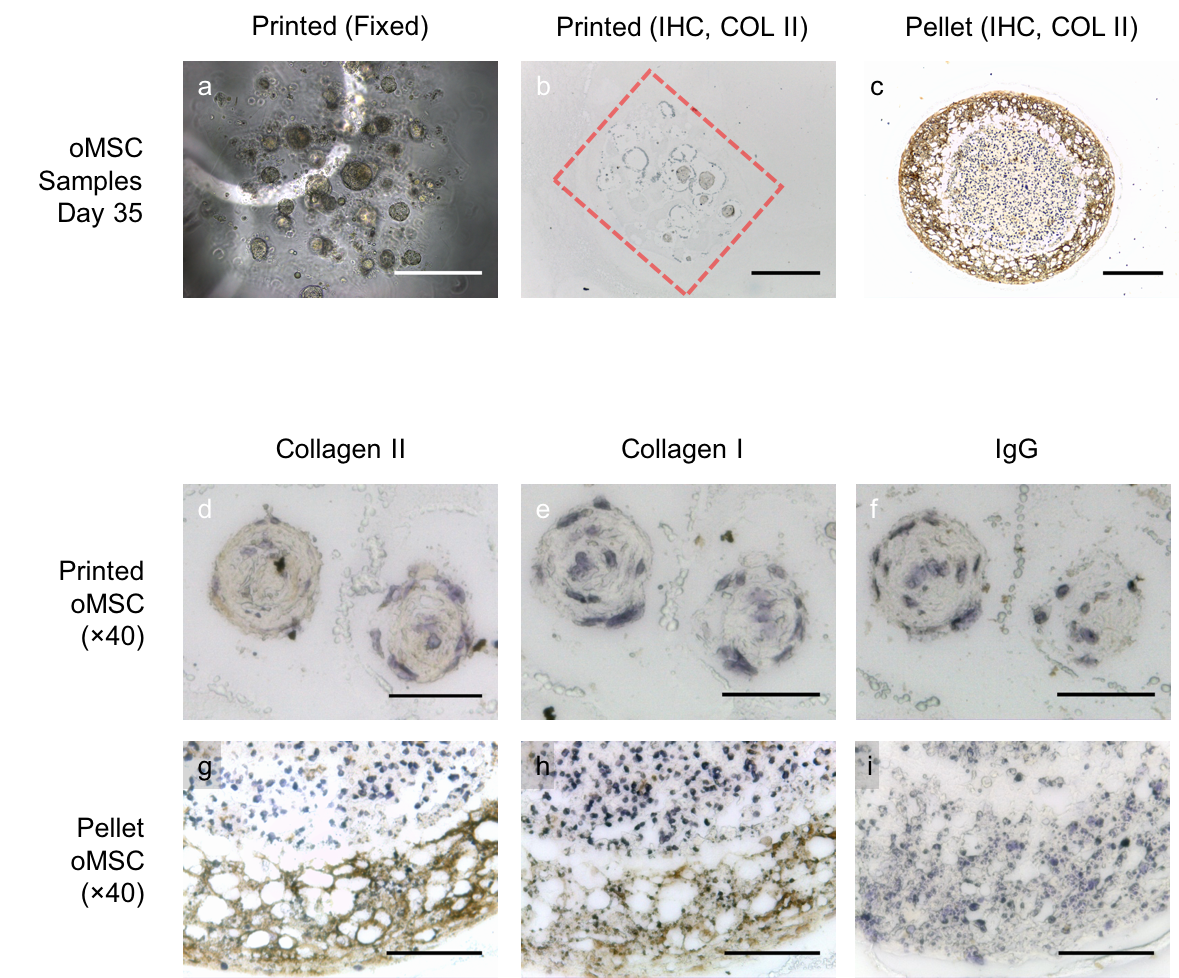


**Supplementary Figure 25**: Immunohistochemistry (IHC) of printed and pellet oMSCs after 35 days of culture with TGF-β3. **a**, Wide-field micrograph of a printed oMSC construct after fixation. **b**-**i**, Wide-field micrographs displaying 4 μm thick sections of a printed oMSC construct and an oMSC pellet sample. The sections were immunoperoxidase stained with diaminobenzidine tetrahydrochloride (DAB, brown) for type I collagen (**e** and **h**) and type II collagen (COL II, **b**, **c**, **d** and **g**). An IgG negative control showed negligible immunostaining (**f** and **i**), and all samples were counterstained with hematoxylin QS (blue). The samples are shown, **a**-**c**, in their entirety and, **d**-**i**, at high magnification. Scale bars are: **a**-**c**, 250 μm; **d**-**f** 50 μm; and **g**-**i** 100 μm.

**Supplementary STR Profiles:**


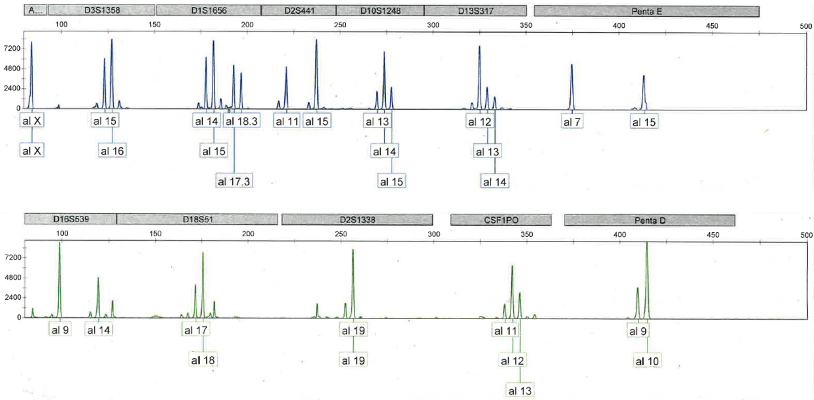


**Supplementary Cell Profile 1**: Section one of two, of the HEK-293T derivative STR profile. For some of the loci more than two alleles are present suggesting either genetic instability or cross contamination with a second independent cell line. As stated in the text this cell line in this study was selected for its generic cellular characteristics.


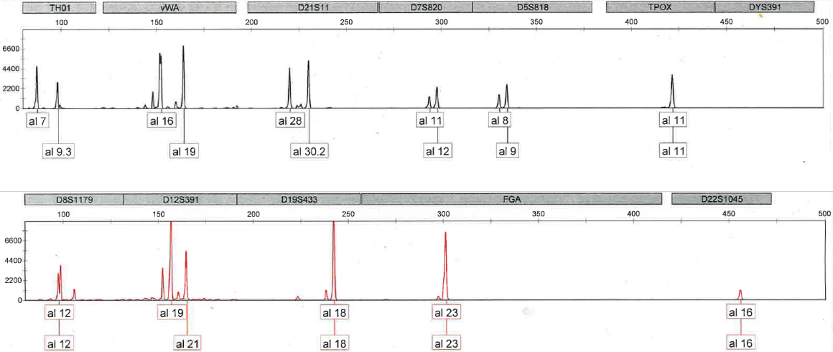


**Supplementary Cell Profile 2**: Section two of two, of the HEK-293T derivative STR profile. For some of the loci more than two alleles are present suggesting either genetic instability or cross contamination with a second independent cell line. As stated in the text this cell line in this study was selected for its generic cellular characteristics.

**Supplementary Information References**

1. Villar, G., Graham, A. D. & Bayley, H. A tissue-like printed material. *Science* **340,** 48–52 (2013).

2. Niu, X. Z. *et al.* Droplet-based compartmentalization of chemically separated components in two-dimensional separations. *Chem. Commun.* **41,** 6159–6161 (2009).

3. Florian, S. & Mayer, T. U. The functional antagonism between Eg5 and dynein in spindle bipolarization is not compatible with a simple push-pull model. *Cell Rep.* **1,** 408–416 (2012).

4. Vytas Bindokas, P. M. White Balance Correction [Image J Plug-in Software, Version 2.0]. (2014).

5. Schindelin, J. *et al.* Fiji: an open-source platform for biological-image analysis. *Nat. Methods* **9,** 676–682 (2012).

6. Schneider, C. A., Rasband, W. S. & Eliceiri, K. W. NIH Image to ImageJ: 25 years of image analysis. *Nat. Methods* **9,** 671–675 (2012).

7. Rasband, W. S. ImageJ [Software, Version 1.49]. (1997).

8. De Vos, K. Cell Counter [ImageJ Plug-in Software, Version 2.0]. (2001).

9. Bolte, S. & Cordelieres, F. P. 3D Object Counter [Image J Plug-in Software, Version 2.0]. (2006).

10. Bolte, S. & Cordelieres, F. P. A guided tour into subcellular colocalisation analysis in light microscopy. *J. Microsc.* **224,** 213–232 (2006).

11. Wolfram-Alpha. Cubic Equation Solver Widget. (2015). Available at: http://www.wolframalpha.com/widgets/view.jsp?id=3f4366aeb9c157cf9a30c90693eafc55.

12. Zhou, M. *et al.* Self-assembled peptide-based hydrogels as scaffolds for anchorage-dependent cells. *Biomaterials* **30,** 2523–2530 (2009).
